# Supplementary material for: EHD1-dependent traffic of IGF-1 receptor to the cell surface is essential for Ewing sarcoma tumorigenesis and metastasis
Source: Commun Biol. 2023 Jul 20;6:758. doi: 10.1038/s42003-023-05125-1 (PMC10359273; doi:10.1038/s42003-023-05125-1)
Supplement: Supplementary file 2 — Supplementary Information [file 42003_2023_5125_MOESM2_ESM.pdf]

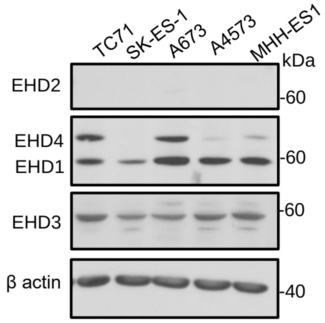

Supplementary Fig. S1. Expression of EHD family proteins in Ewing Sarcoma cell lines. Shown is an immunoblot analysis of EHD family members in the indicated EWS cell lines. β actin served as loading control.

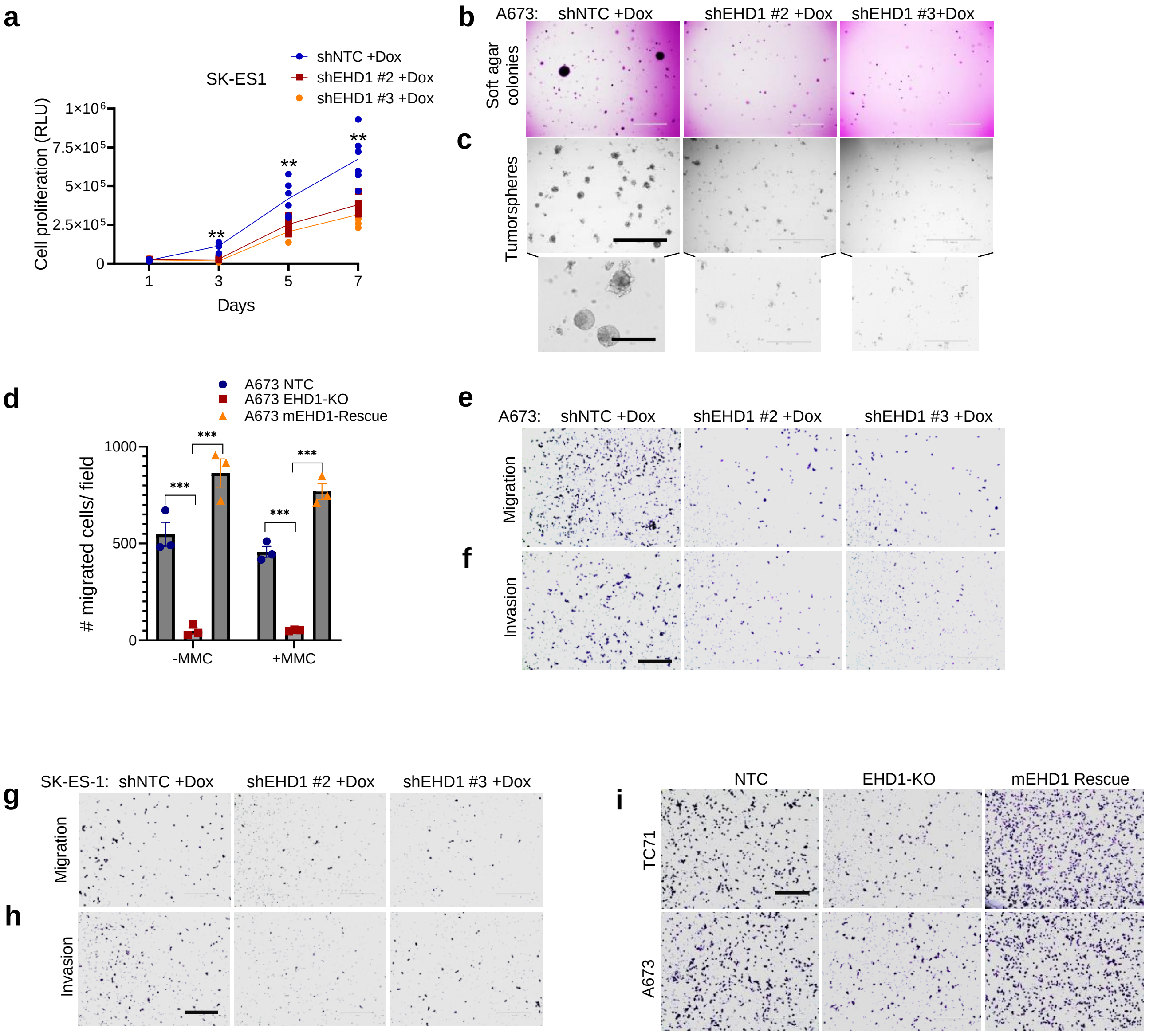

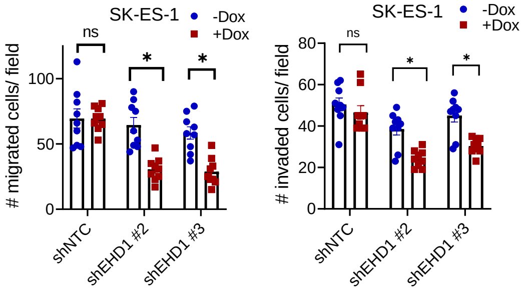

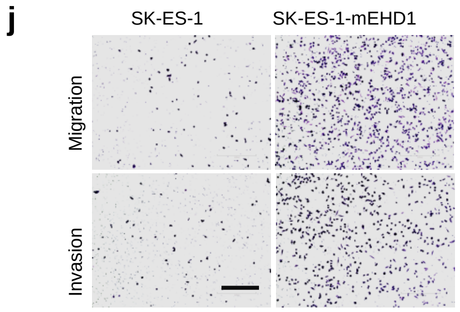

Supplementary Fig. S2. EHD1 is required to sustain the in vitro oncogenic traits of Ewing Sarcoma cell lines (a) Impaired proliferation of SK-ES-1 cells upon EHD1 knockdown. Cell-titer-glo assay at the indicated time points in the presence of Dox. Mean +/- SEM of three experiments, each with six replicates. **(b-c)** Impaired soft agar colony growth (b) and tumor-sphere forming ability (c) of A673 cells upon EHD1 knockdown. Representative images of A673 cells (Fig. 2E-F; bottom) are shown. **(d)** Treatment with Mitomycin C (10 µg/ml) to show the negligible role of cell proliferation on cell migration assay in A673 NTC, EHD1-KO and mEHD1 Rescue cell lines. **(e-f)** Impaired trans-well migration (e) and invasion (f) in A673 cells upon EHD1 knockdown. Representative images of A673 cells (Fig. 2g-h; bottom) are shown. **(g-h)** Impaired of trans-well migration (g) and invasion (h) in SK-ES-1 cells upon EHD1 knockdown. Top, representative images of SK-ES-1 cells; scale bar, 400 µm. Bottom, quantification of the number of migrated/invaded cells per high-power field; mean +/- SEM of three experiments each in triplicates. **(i)** Representative images of Fig2i showing impaired trans-well cell migration upon EHD1 knockout (KO) and rescue of migration defect by mEHD1. scale bar, 400 µm **(j)** Representative images of Fig2l-m showing increase in trans-well migration and invasion upon mEHD1 overexpression in SK-ES-1 cells, scale bar, 400 µm.

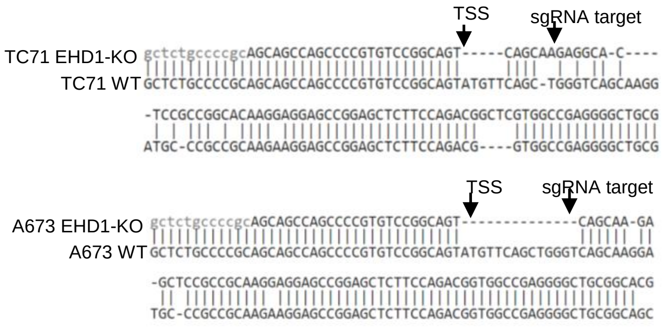

Supplementary Fig. S3. Verification of CRISPR-Cas9 mediated *EHD1* gene targeting in EWS cell lines. CRISPR-Cas9 knockout sites in *EHD1* gene were assessed by Sanger sequencing of genomic DNA PCR fragments corresponding to the targeted region (see Methods). Shown is the deletion of bases, removal of the start codon and the frameshift mutations near the sgRNA targeted sequence in TC71 and A673 cell lines. Wildtype (WT) sequences are shown as reference.

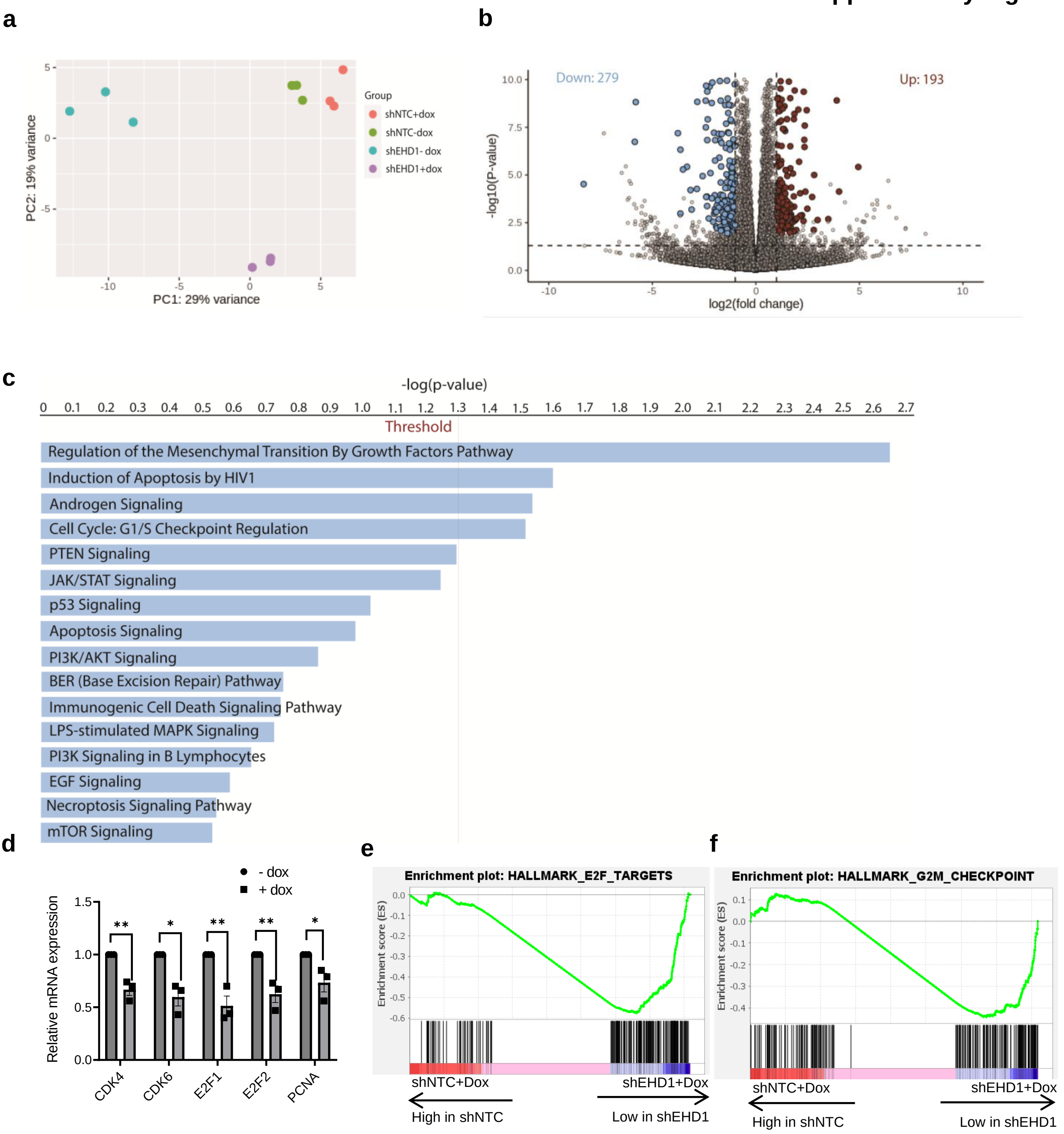

Supplementary Fig. S4. RNA-Sequencing analysis of shNTC and shEHD1+dox TC71 cell line (a) PCA analysis of RNA-seq data shows four datasets – TC71 shNTC -/+Dox, shEHD1 -/+ Dox. PC1 represents 29% variance and PC2 represents 19% variance. **(b)** Volcano plot showing differentially expressed genes – upregulated (in red), downregulated (in blue). **(c)** Canonical signaling pathways affected by the differentially expressed genes by Ingenuity-Pathway Analysis (IPA) software. Vertical line indicates threshold of $-\log_{10}$(p-value) =1.3 **(d)** Validation of G1 to S cell cycle regulatory genes by qPCR analysis in TC71 shEHD1 -/+Dox groups. (mean +/- SEM of three experiments, *p<0.05, **p<0.01) **(e-f)** Gene-set enrichment (GSE) analysis was performed on the RNA-sequencing of two groups of TC71 cell lines- TC71 shEHD1+Dox vs. shNTC+Dox, showing enrichment of E2F targets (e) and G2-M cell cycle checkpoint(f) genes in shNTC+Dox cells and significant downregulation of the same in the shEHD1+Dox group. Differential expression was assessed by DESeq2 and significantly changed genes were required to have a Benjamini–Hochberg adjusted p-value of <0.05 and a 2-fold change in expression.

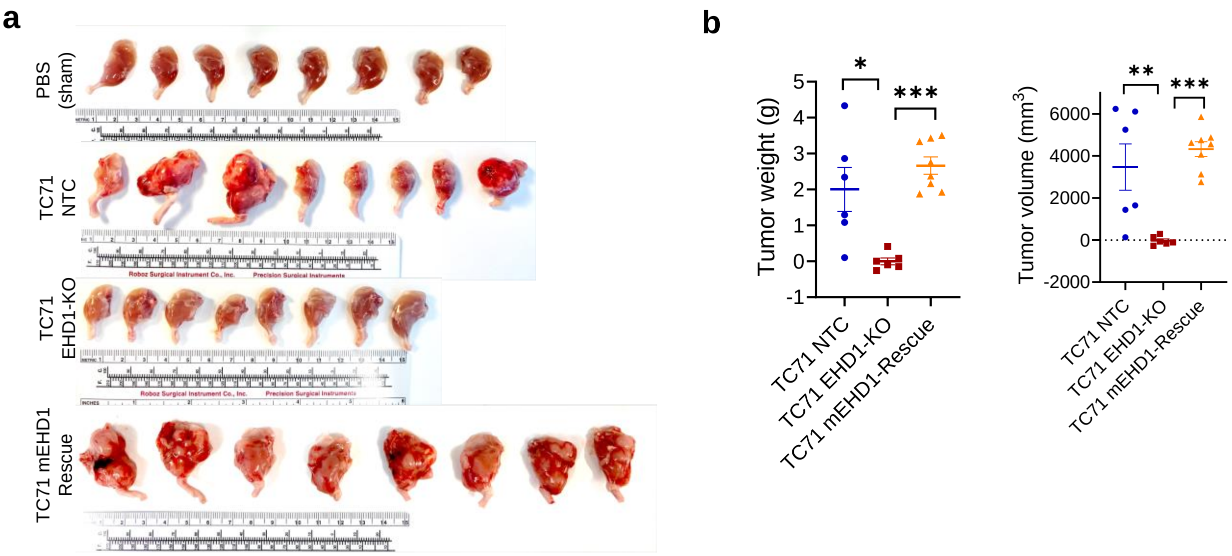

Supplementary Fig. S5. Loss of EHD1 expression markedly impairs the growth of bone implanted EWS cells. (a) Images of tumors harvested at the end of the experiment shown in Fig. 3A-D together with the sham (PBS)-injected contralateral legs of the mice injected with TC71-NTC cells. **(b)** Quantification of harvested tumor weight (left panel) and volume (measurement with calipers (volume = length x width x depth/2); right panel. The values of sham-injected legs were subtracted from the experimental values.

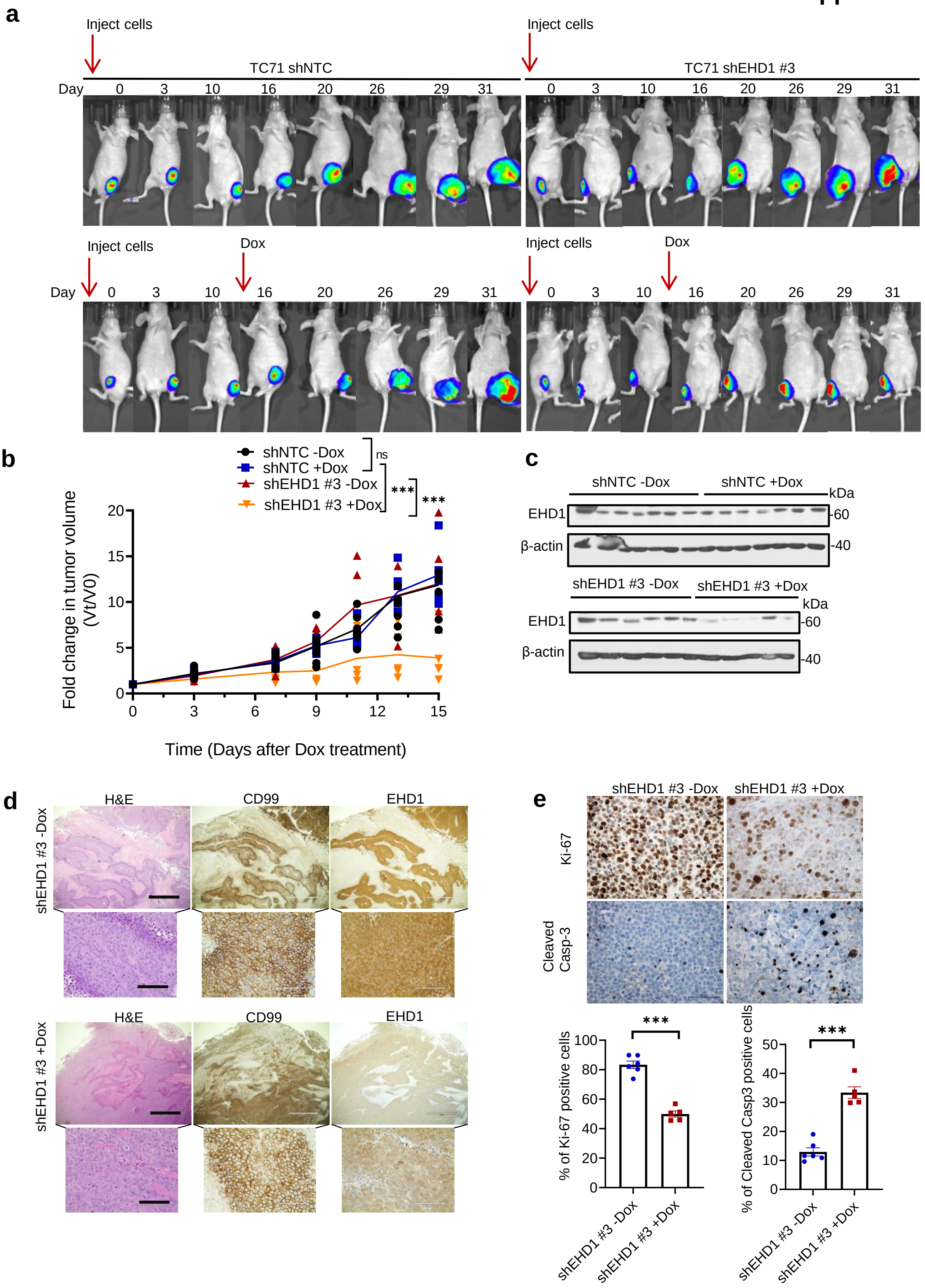

Supplementary Fig. S6. Demonstration of EHD1 requirement for EWS tumorigenesis using Dox-inducible shRNA knockdown. Intratibial tumor injections with the indicated TC71 cell lines with Dox-inducible control (shNTC) or EHD1 (shEHD1#3) shRNA were done as in Fig. 3.; 7 mice/group. **(a)** Images of one out of seven mice in various groups with super-imposed luminescence signals over 31 days. **(b)** Tumor growth with the injections of the indicated TC71 derivatives, with or without Dox administration. Differences between the indicated groups analyzed using the two-way ANOVA; ***p<0.001. Note lack of impact of Dox on tumors generated with TC71 shNTC. **(c)** Western blots of harvested tumor tissue to confirm Dox-induced EHD1 knockdown in TC71 shEHD1 #3 xenografts. **(d)** Representative tumor sections of TC71-shEHD1-Dox and shEHD1+Dox tumors stained with H&E (left panels), CD99 (middle panels, demarcating the human EWS tumor cell area) and EHD1 (right panels). **(e)** IHC staining for Ki-67 and Cleaved-Caspase-3 in tumor sections from the indicated groups. Top, representative images; bottom, quantification IHC staining positive cells. Mean +/- SEM; *p<0.05, **p<0.01, ***p<0.001, ns= not significant.

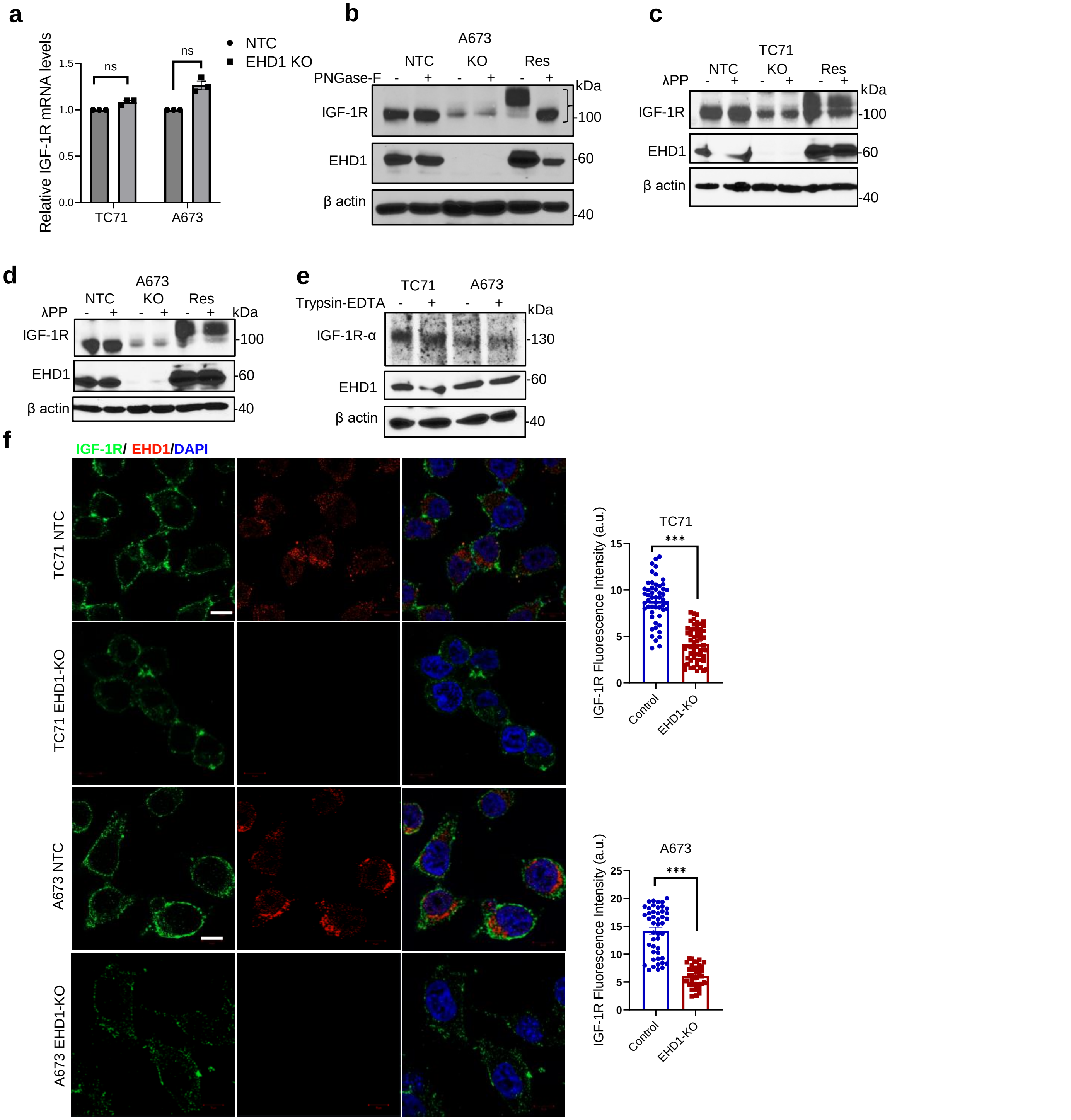

Supplementary Fig. S7. Identification of insulin like growth factor-1 receptor (IGF-1R) as a regulatory target of EHD1 in EWS. (a) EHD1-KO in EWS cell lines does not affect the IGF-1R mRNA expression. Shown are the qRT-PCR based results of IGF-1R mRNA expression, normalized to GAPDH and expressed as a fold change relative to the respective NTC control cell lines (set to 1). Data represents mean +/- SEM of 3 independent experiments (ns= not significant). **(b)** Slower migration of IGF-1R band in immunoblots of exogenous mEHD1 rescue/overexpressing A673 is due to increased N-linked glycosylation. Indicated cell lines were left untreated or treated with PNGase-F (10,000 U/ml for 30 minutes) followed by anti-IGF-1R immunoblotting. β actin served as loading controls. **(c-d)** Slower migration of IGF-1R in immunoblots of exogenous mEHD1 rescue/overexpressing EWS cell lines is not due to increased phosphorylation. Indicated cell lines were left untreated or treated with Lambda-phosphatase(30 minutes in a 50 µl reaction) followed by anti-IGF-1R immunoblotting. β actin served as loading controls. **(e)** Trypsin-EDTA release of cells used for FACS analysis does not result in degradation of IGF-1R. Lysates of the indicated cell lines were prepared either directly or after Trypsin EDTA treatment and washing as used for FACS analyses of the cell surface IGF-1R and immunoblotted with an anti-IGF-1R α antibody. Comparable signals are seen in direct lysates vs. lysates prepared after trypsin-EDTA treatment. **(f)** Reduction in IGF-1R levels upon EHD1-KO in EWS cell lines analyzed by immunofluorescence staining and confocal imaging. IGF-1R (green) and EHD1 (red) staining in Control and EHD1-KO TC71 and A673 cells. Cells grown under steady-state were fixed and permeabilized and stained with the indicated antibodies (with concurrent IgG controls; not shown). Left, representative confocal images. Merged pictures with DAPI (blue) are shown in right panels. Right, Quantification of the IGF-1R fluorescence intensity. Scale bar (only shown in left panels of the NTC lines), 10 µm. Data points represent images of 60 cells pooled from three independent experiments.

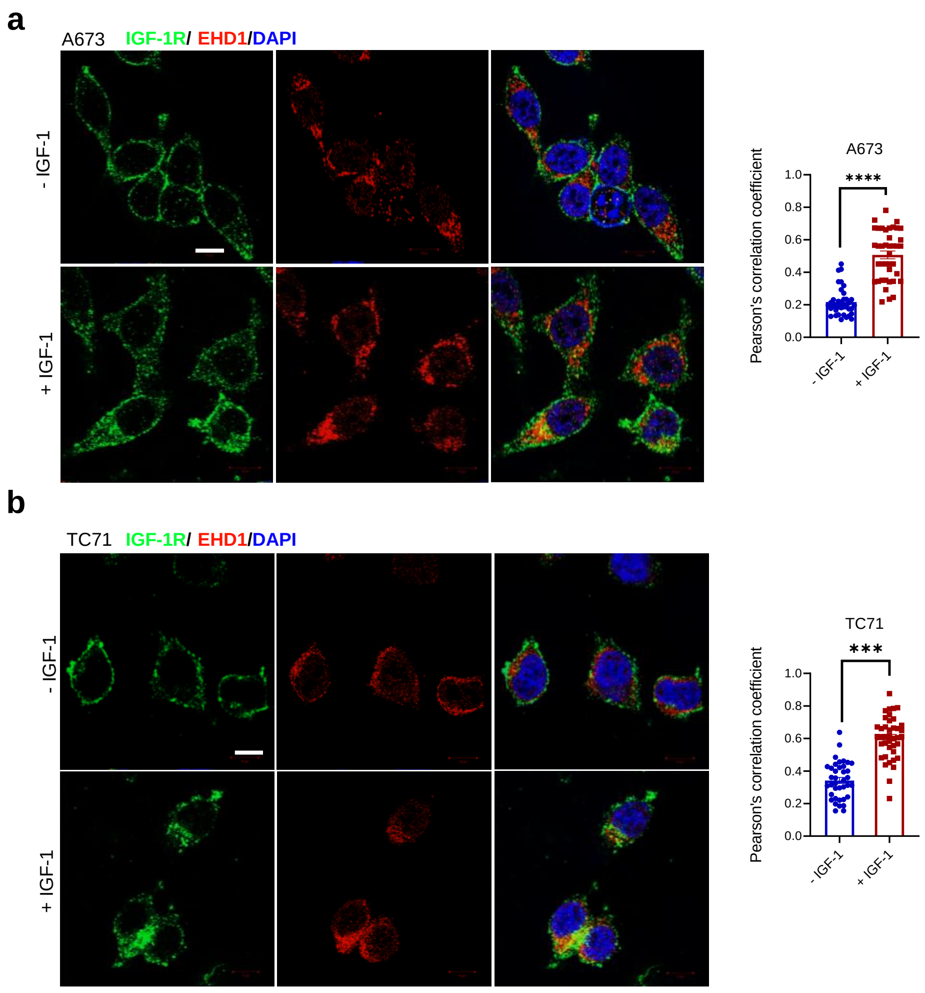

Supplementary Fig. S8. EHD1 and IGF-1R colocalize in intracellular vesicular structures. (a-b) Representative confocal images of the colocalization of EHD1 (red) and IGF-1R (green) in A673 (a) and TC71 (b) cells without (top panels) and with (bottom panels) IGF-1 (50 ng/ml) stimulation for 1h. Merged pictures (right panels) with DAPI (blue) show colocalization within perinuclear vesicular structures. Scale bar, 10 μm. Colocalization was assessed in 40 cells in three independent experiments to determine the colocalization coefficients. Data represent the mean +/- SEM. ***p<0.001.

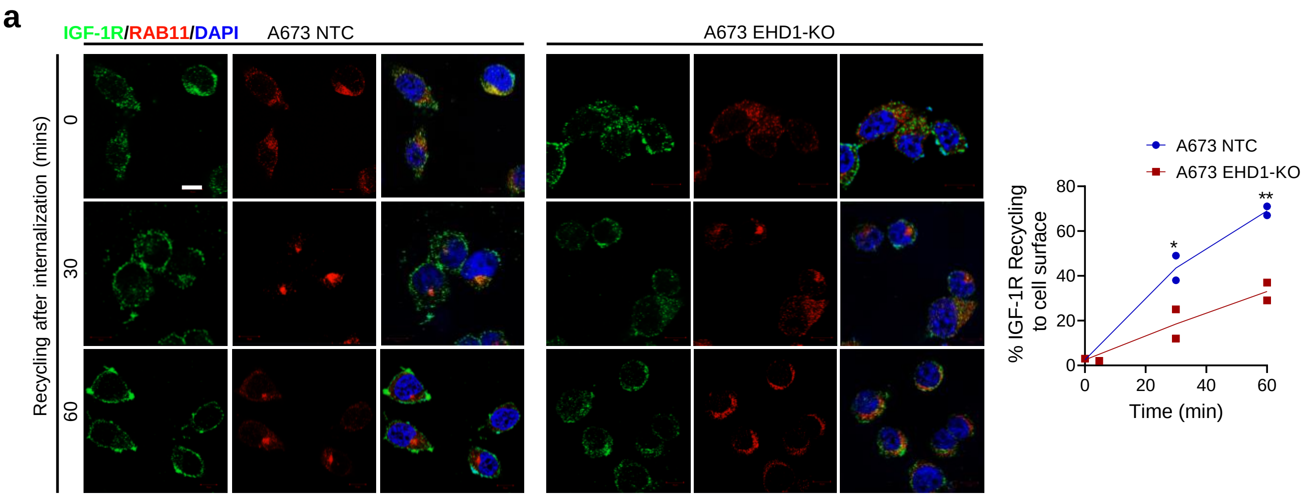

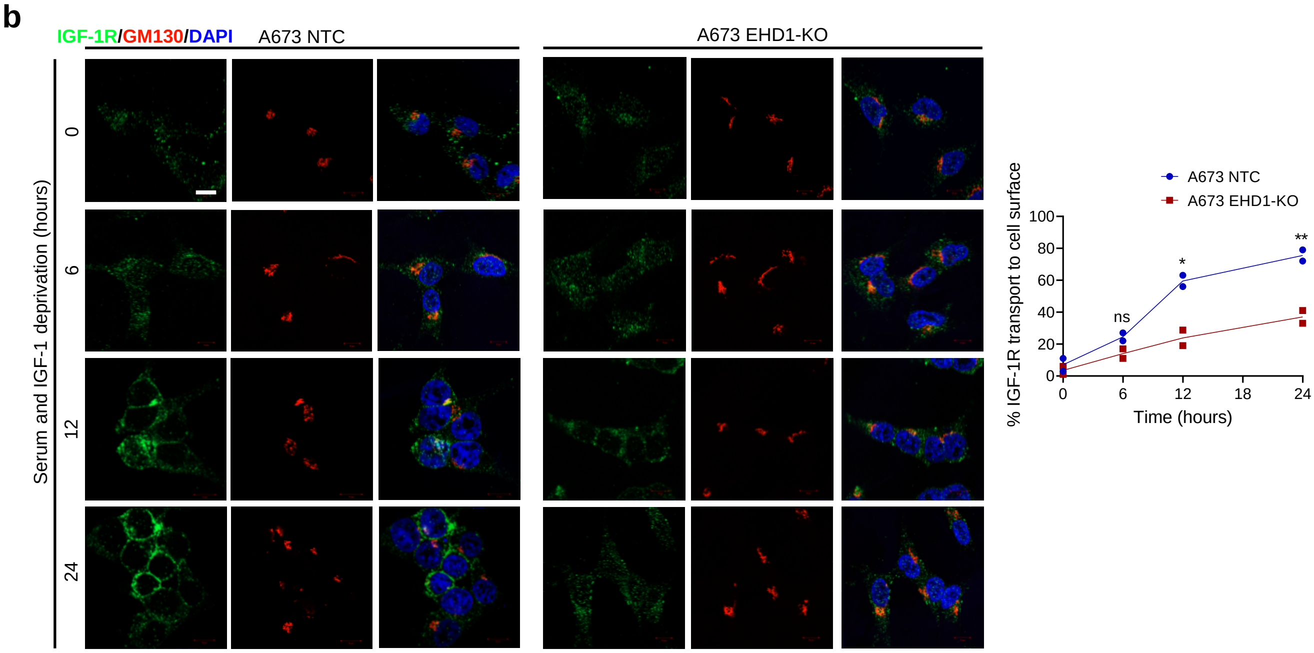

Supplementary Fig. S9. Impairment of IGF-1R transport from the Golgi and recycling endosomes to the plasma membrane by EHD1-KO in A673 cell line. The analyses with A673 NTC and EHD1-KO cells were carried out exactly as described in Fig. 5 for TC71 cells. **(a)** Analysis of IGF-1R endocytic recycling; IGF-1R, green; Recycling endosome (Rab11+), red. **(b)** Analysis of IGF-1R Golgi to plasma membrane transport; IGF-1R, green; Golgi (GM130+), red. Left, representative confocal images right, quantification of cell surface IGF-1R at various time points using ImageJ. Data represent mean +/- SEM. *p<0.05; **p<0.01; ns, not significant. Scale bar, 10 μm.

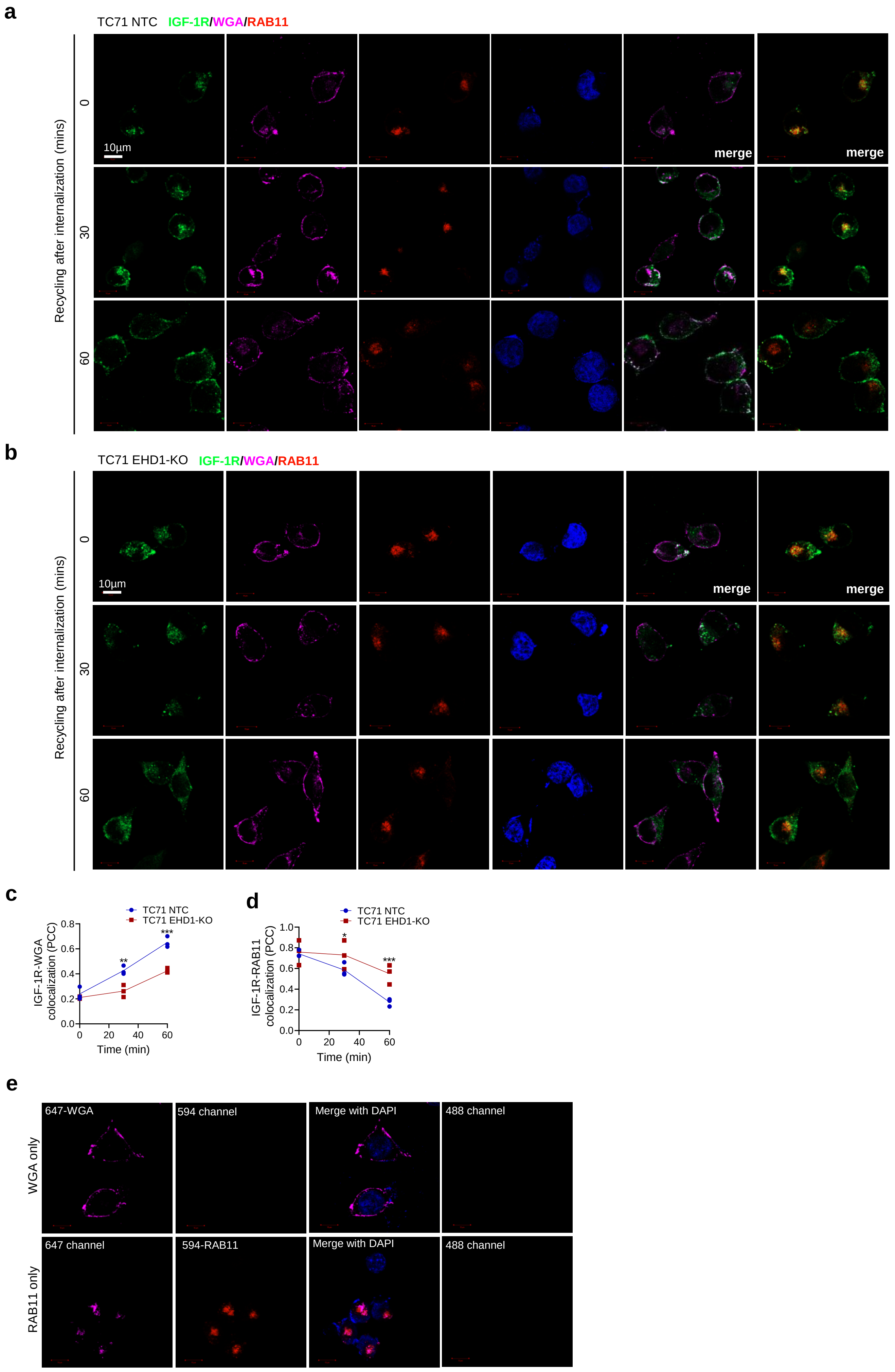
a
TC71 NTC IGF-1R/WGA/RAB11
Recycling after internalization (mins)
0
30
60
10µm
merge
merge
b
TC71 EHD1-KO IGF-1R/WGA/RAB11
Recycling after internalization (mins)
0
30
60
10µm
merge
merge
c
TC71 NTC
TC71 EHD1-KO
IGF-1R-WGA colocalization (PCC)
0.0
0.2
0.4
0.6
0.8
0
20
40
60
Time (min)
**
***
d
TC71 NTC
TC71 EHD1-KO
IGF-1R-RAB11 colocalization (PCC)
0.0
0.2
0.4
0.6
0.8
1.0
0
20
40
60
Time (min)
*
***
e
WGA only
RAB11 only
647-WGA
594 channel
Merge with DAPI
488 channel
647 channel
594-RAB11
Merge with DAPI
488 channel

Supplementary Fig. S10. Confirmation of the impaired endocytic recycling compartment to plasma membrane transport of IGF-1R upon EHD1-KO in TC71 cell line by colocalization with a plasma membrane marker. The IGF-1R recycling assays with NTC and EHD1-KO TC71 cells were carried out as described in main Fig. 5b, with additional staining of cells prior to permeabilization with the plasma-membrane marker wheat germ agglutinin (WGA). **(a-b)** Representative images of co-staining of IGF-1R (green), WGA; plasma membrane (magenta); Rab11 (red; Endocytic Recycling Compartment); and DAPI (blue; Nuclei). Merged pictures are shown in right panels. Scale bar, 10 μm. **(c-d)** Quantification of the WGA- (c) and Rab11-colocalizing (d) (plasma membrane and Endocytic Recycling Compartment-associated) pools of IGF-1R over various times of chase. Analyses of Pearson's correlation coefficient quantification using ImageJ. 50 cells each from three independent experiments were analyzed. Data represents mean +/- SEM, $^*p<0.05$; $^{**}p<0.01$; $^{***}p<0.001$. **(e)** Staining controls (only TC71-NTC cells cells are shown; others showed comparable results). Shown are the images of cells stained only with WGA (top panels) or only with Rab11 (bottom panels) and co-stained with DAPI, and visualized in the indicated (647, 594, 488 and DAPI) channels. Note that WGA-647 shows no bleed-through in 594 (used for Rab11) or 488 (used for IGF-1R) channels. In contrast, Rab11-594 shows bleed-through into 647 channel (which accounts for magenta signals in WGA staining in a-b) but not in 488 channel. The combinations used were necessitated by the availability of the corresponding fluorescently-conjugated reagents. The colocalization with IGF-1R is based on merging of the corresponding two fluorescence channels.

Supplementary Fig. S11

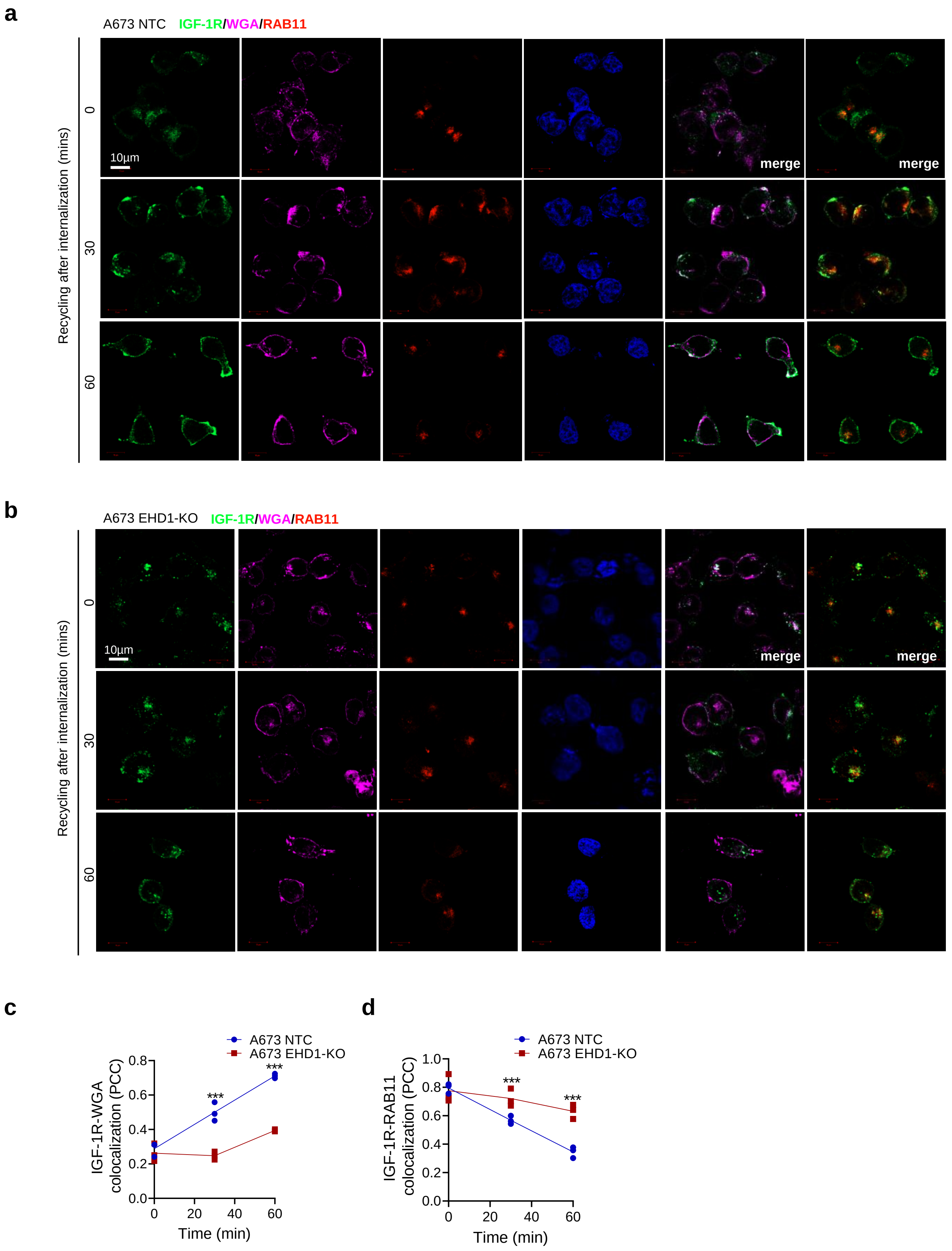

Supplementary Fig. S11. Confirmation of the impaired endocytic recycling compartment to plasma membrane transport of IGF-1R upon EHD1-KO in A673 cell line by colocalization with a plasma membrane marker. The analyses using the indicated A673 cell lines were carried out exactly as in Supplementary Fig. S10. Quantified data in c-d represents mean +/- SEM of three experiments. *p<0.05; **p<0.01; ***p<0.001. Scale bar, 10 µm.

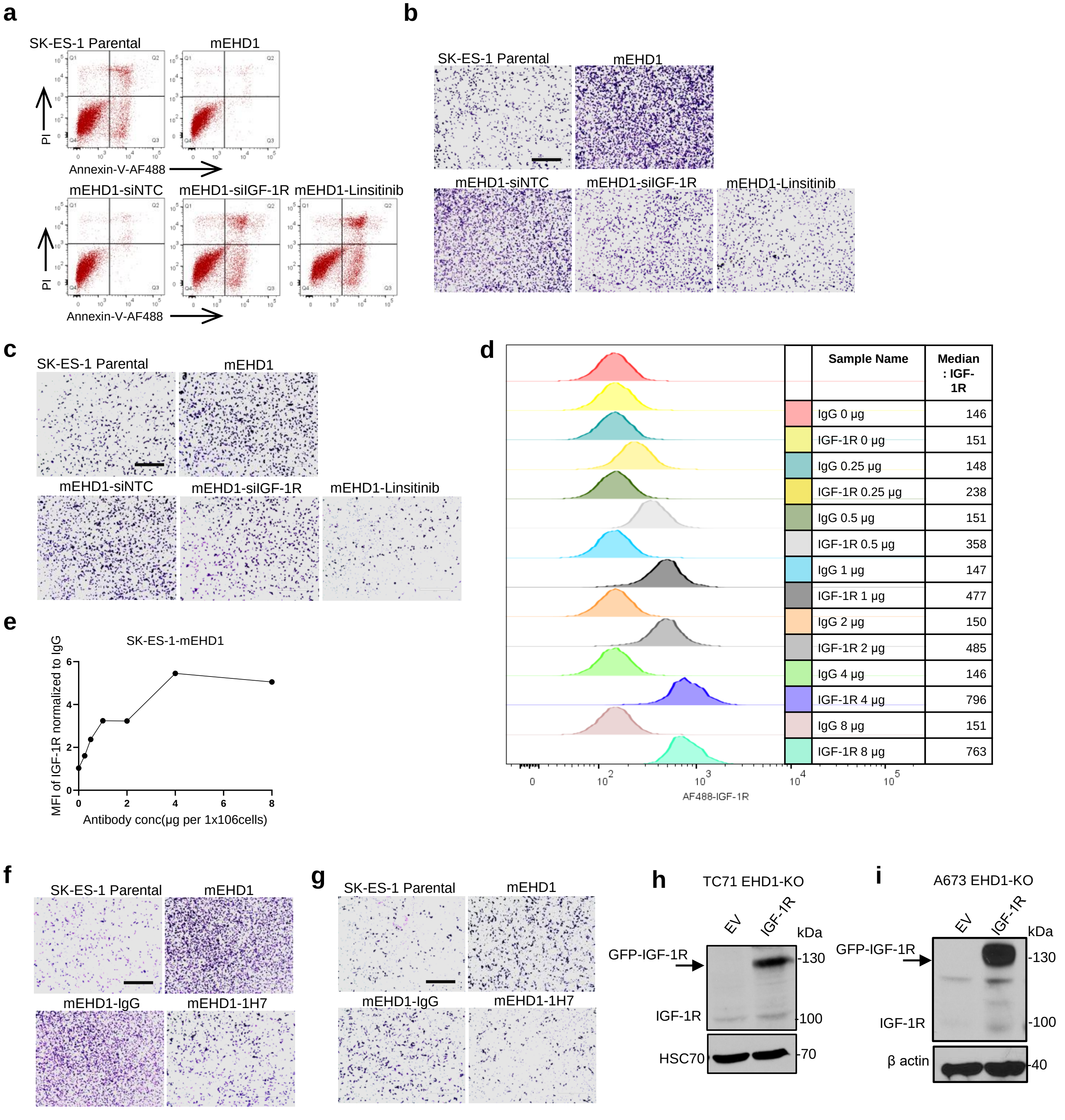

Supplementary Fig. S12. EHD1-dependent upregulation of oncogenic attributes of EWS cell lines requires the IGF-1R. **(a)** Representative flow panel of Annexin-V-PI assay (Figure 8d) in SK-ES-1 mEHD1 overexpressing cell line, with the indicated treatments. **(b-c)** Representative high-power fields of migration and invasion assays corresponding to Figure 8e-f. scale bar, 400 µm. **(d-e)** Dose-response of IGF-1R monoclonal antibody 1H7 showing saturation at 4µg antibody concentration/million cells. Representative flow panels(d), graph plotting Median fluorescence intensity (MFI) normalized to same concentration of mouse isotype control IgG1(e). **(f-g)** Representative high-power fields of migration and invasion assays corresponding to Figure 8h-i. scale bar, 400 µm.**(h-i)** TC71 and A673 EHD1-KO cells were transfected with Empty vector (EV)-GFP or IGF-1R-GFP and selected with selection marker, G418. Representative western blot confirming the effective transfection.

Uncropped western blots: Figure 2

Supplementary Fig. S13

Figure 2a: anti-EHD1 (60 kDa)

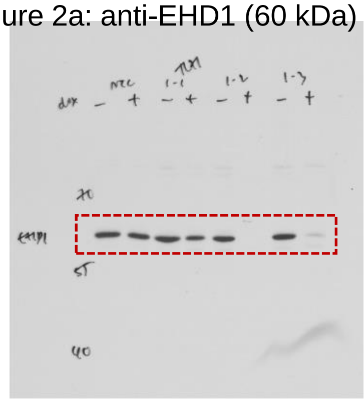

Figure 2a: anti-β-actin (42 kDa)

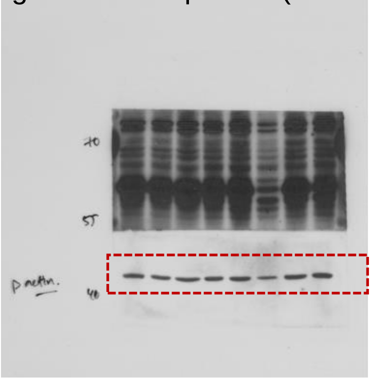

Figure 2a: anti-EHD1 (60 kDa)

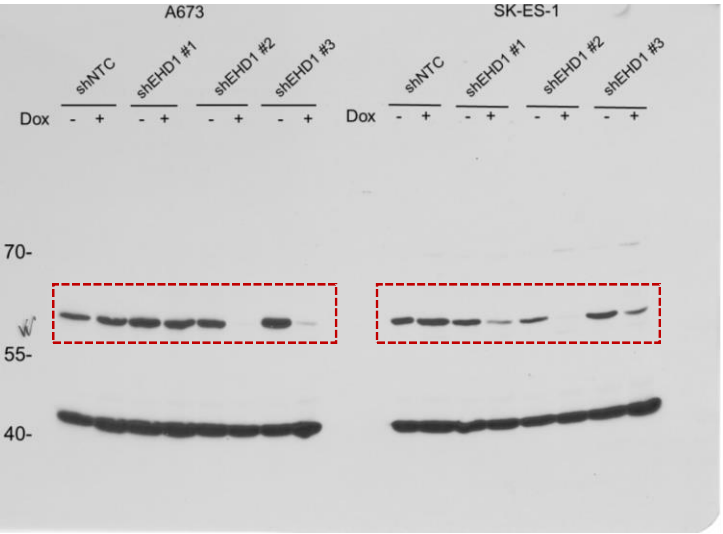

Figure 2a: anti-β-actin (42 kDa)

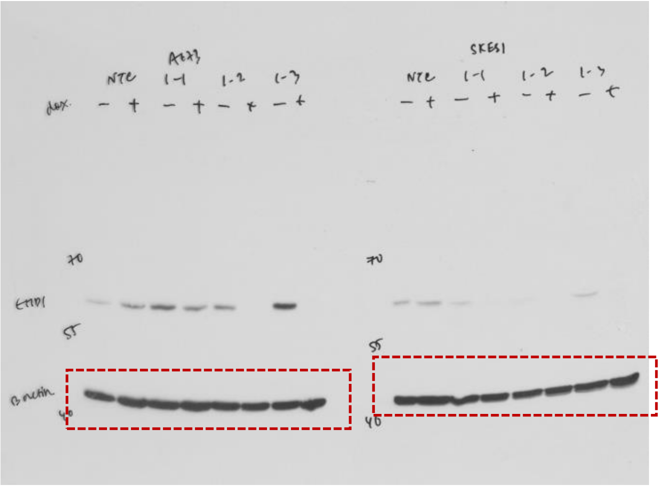

Figure 2b: anti-EHD1 (60 kDa)

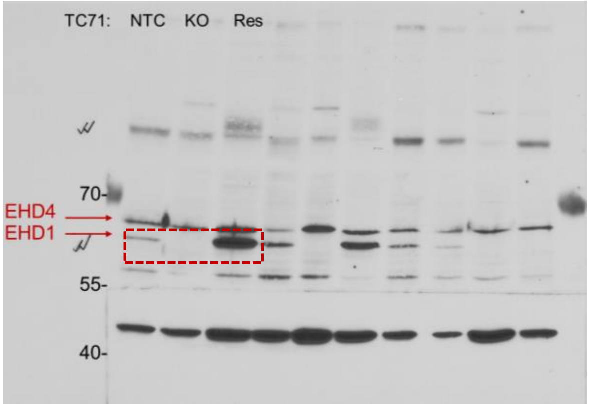

Figure 2b: anti-HSC70 (70 kDa)

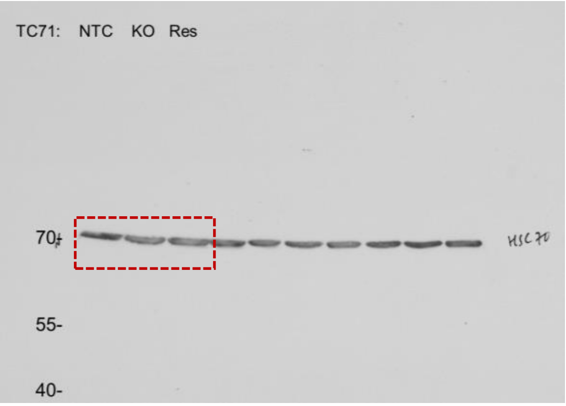

Figure 2b: anti-EHD1 (60 kDa)

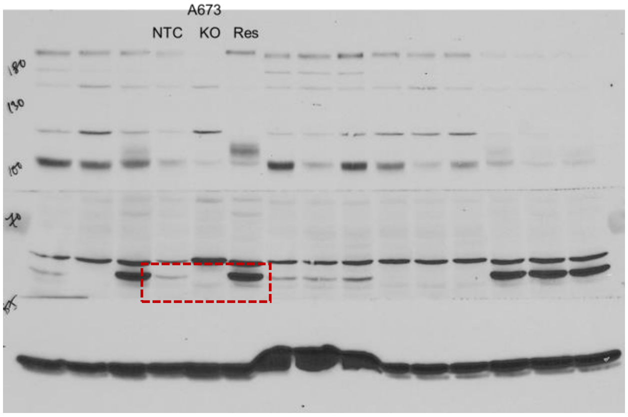

Figure 2b: anti-HSC70 (70 kDa)

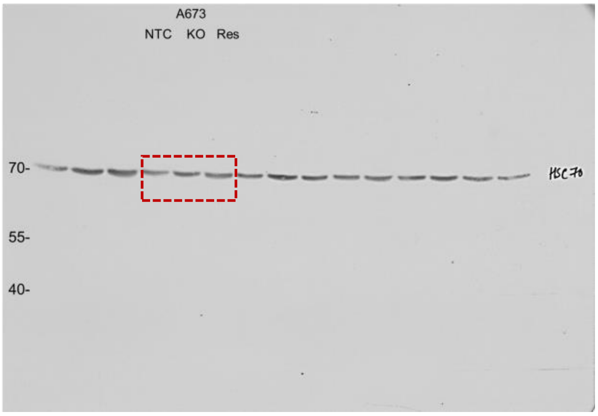

Figure 2

Figure 2j: anti-EHD1 (60 kDa)

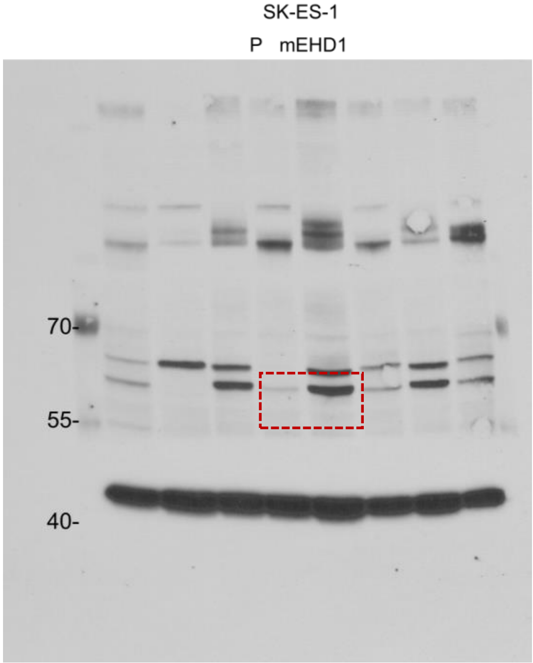

Figure 2j: anti-β-actin (42 kDa)

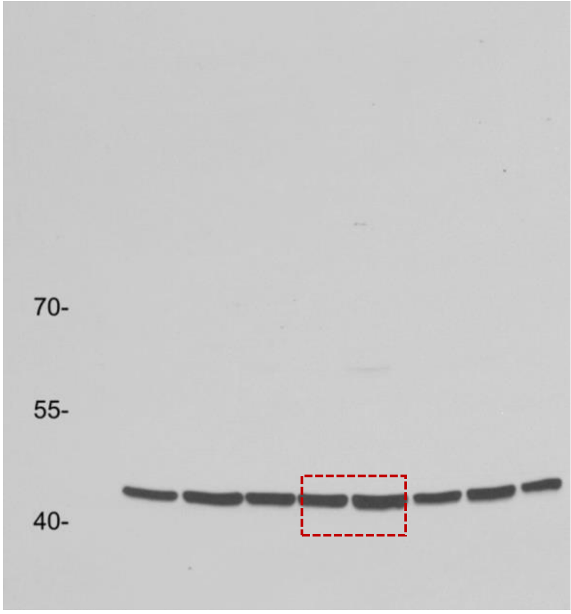

Figure 4

Figure 4b

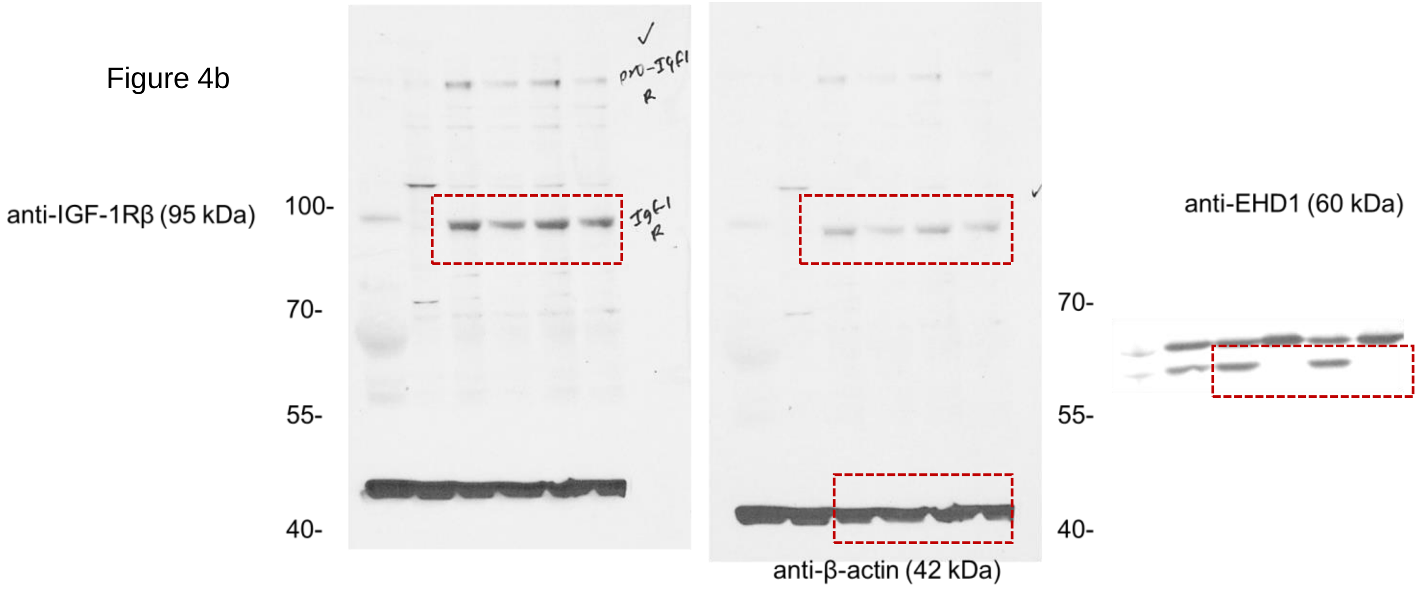

Figure 4c

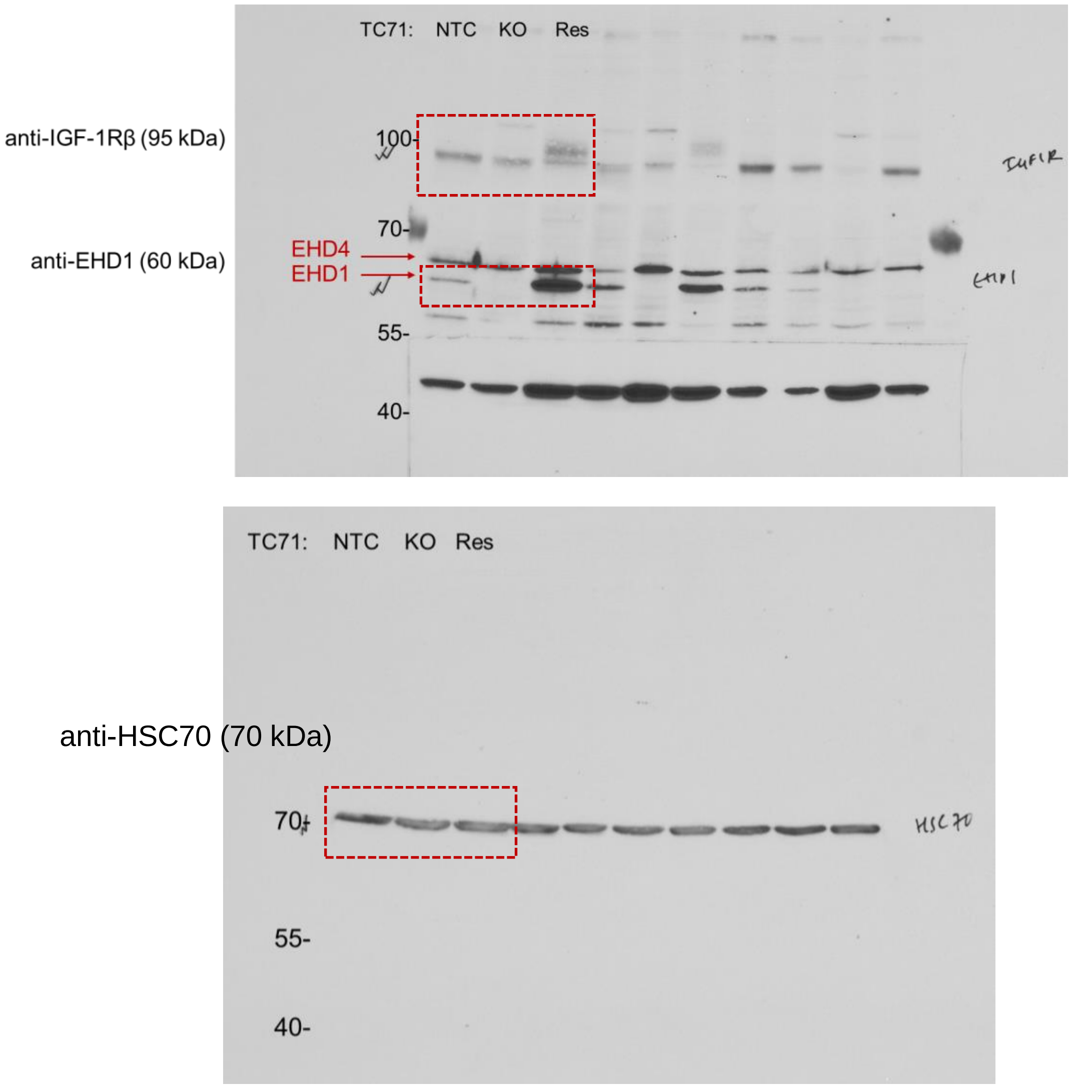

Figure 4

Figure 4c

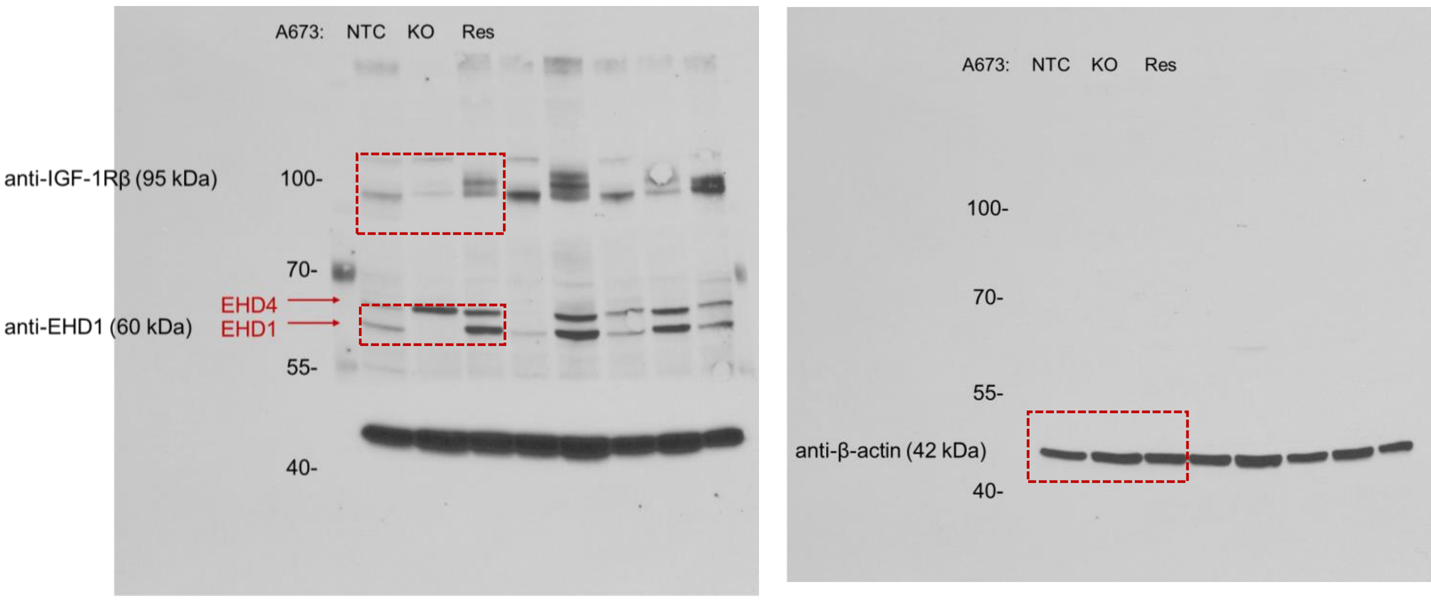

Figure 4d

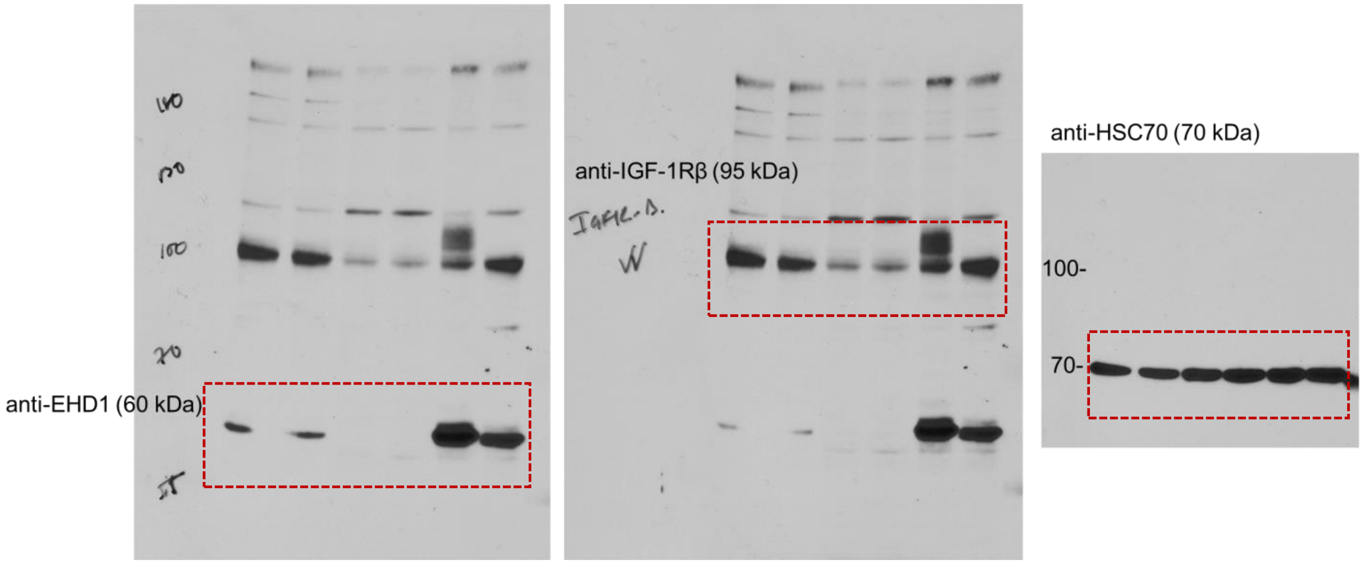

Figure 4e

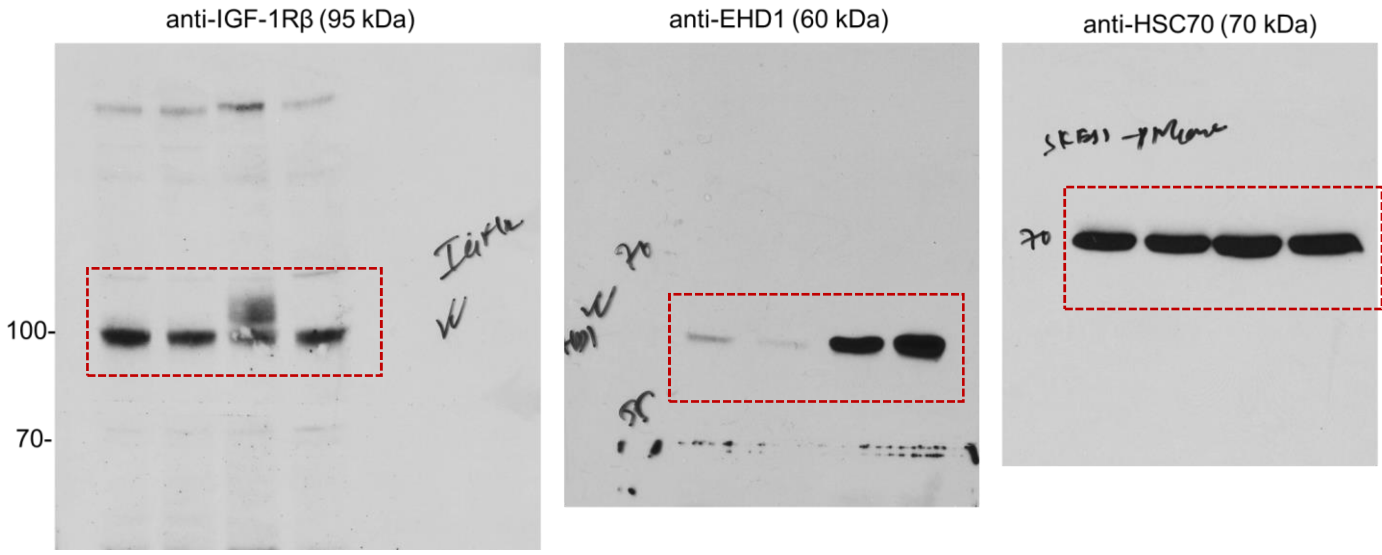

Figure 4

Figure 4i

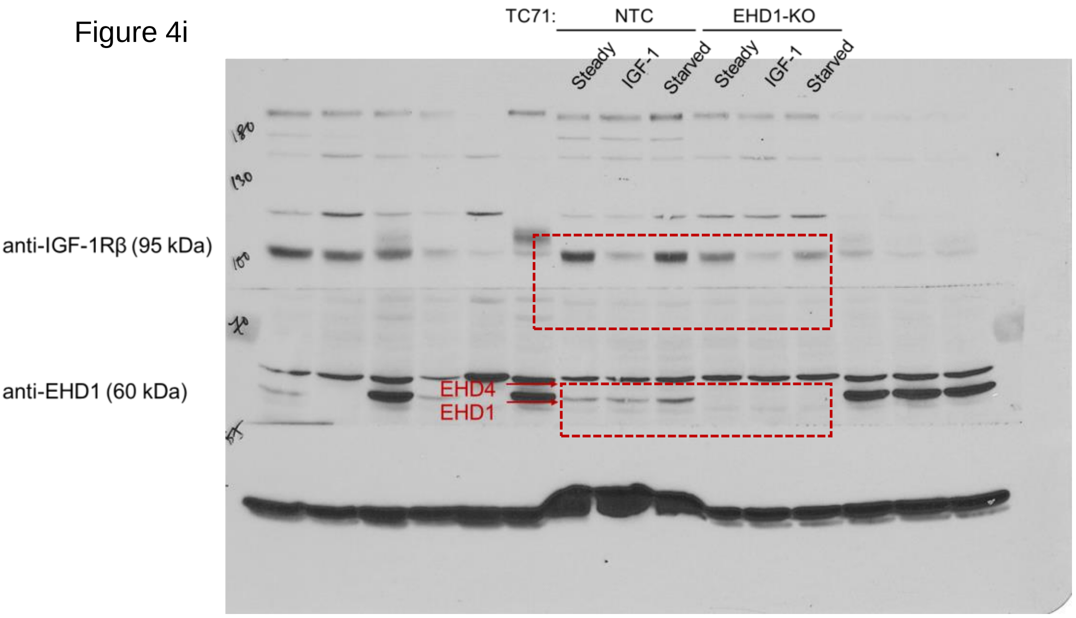

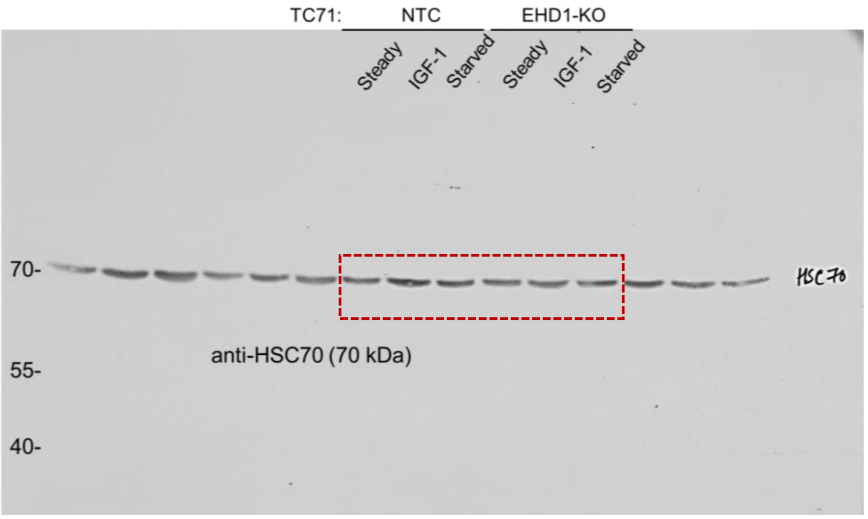

Figure 4

Figure 4i

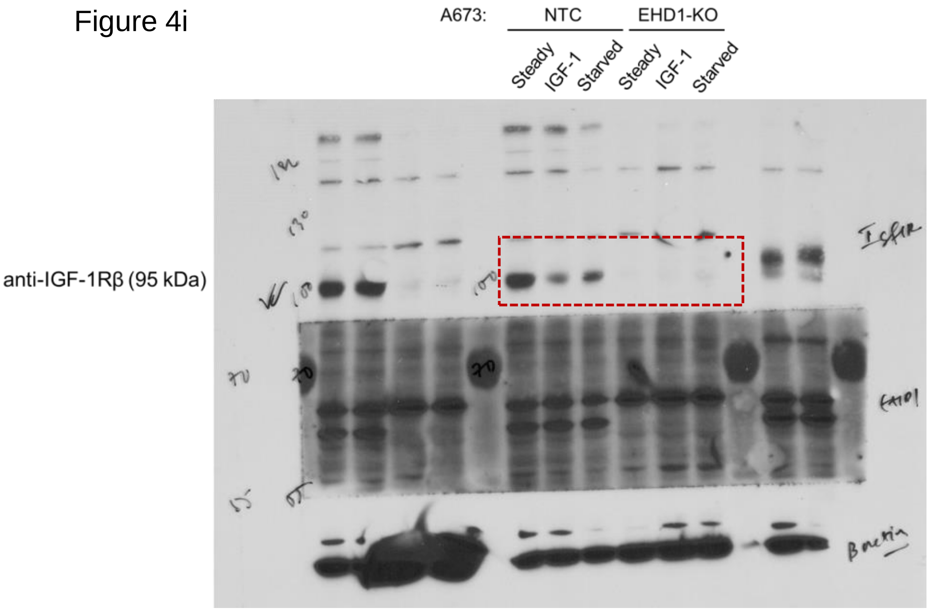

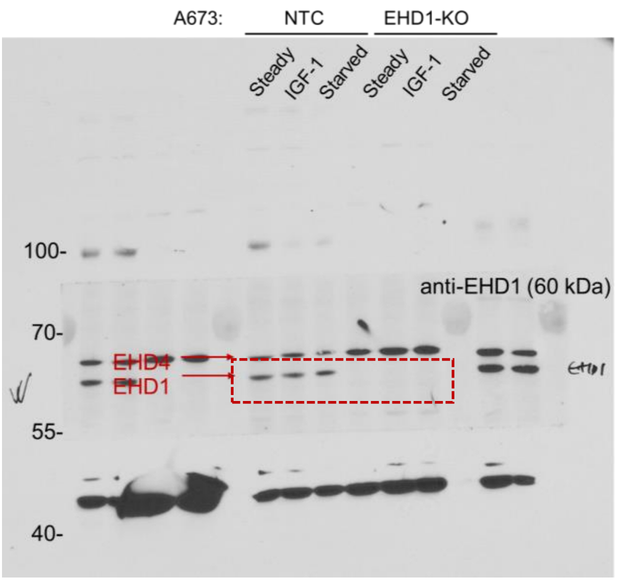

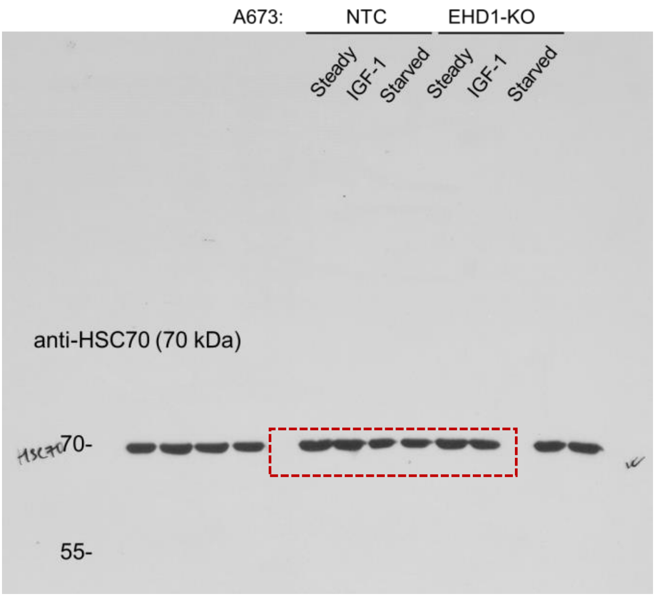

Figure 4j

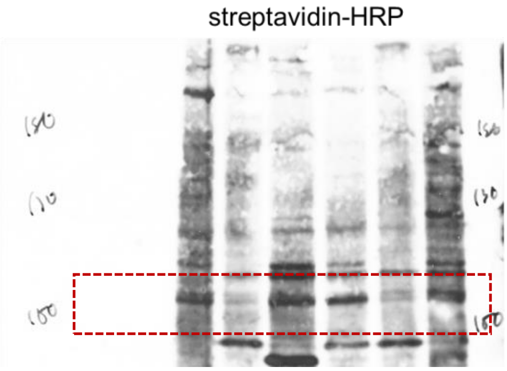

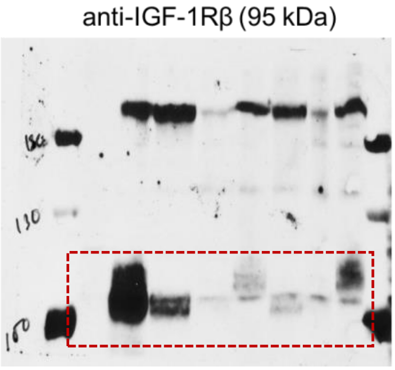

Figure 5

Figure 5a(TC71):

anti-IGF-1Rβ (95 kDa)

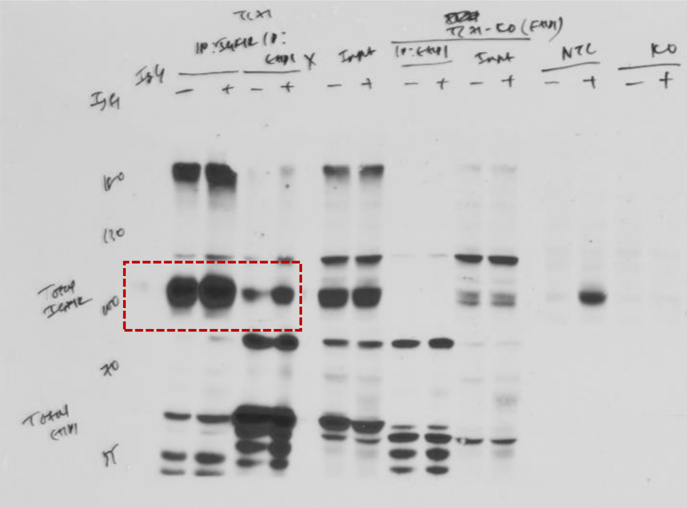

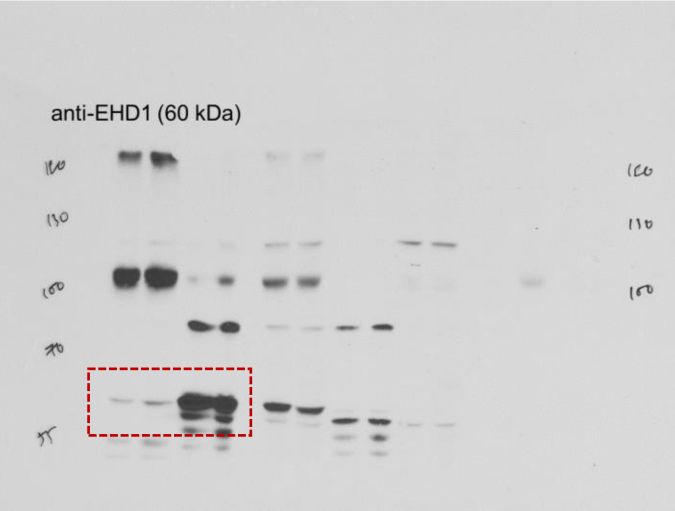

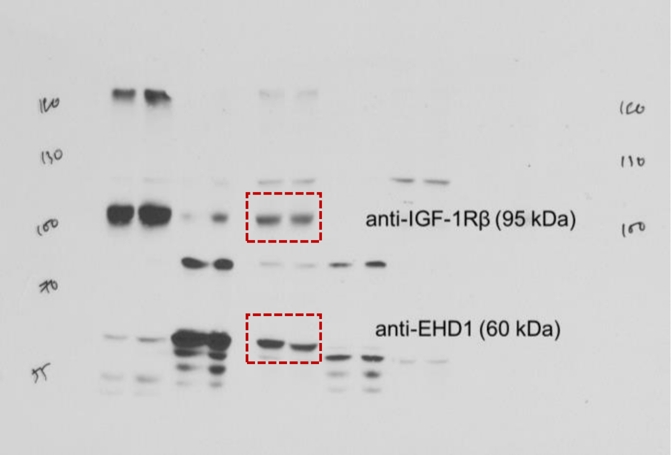

Figure 5a(A673):

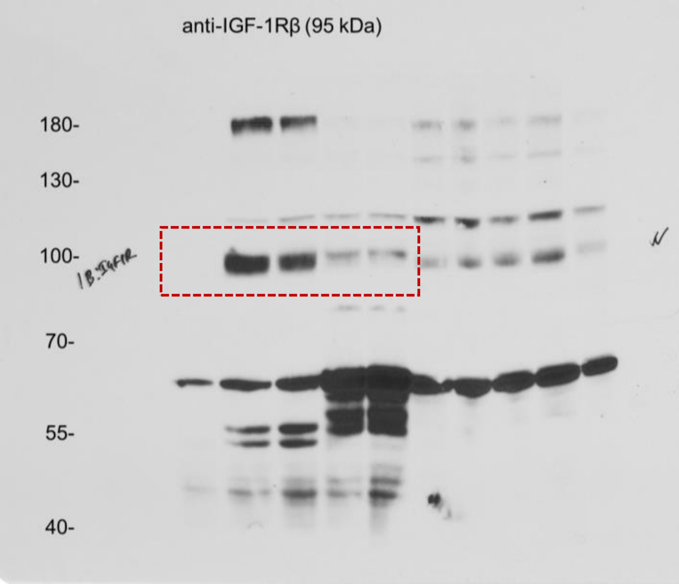

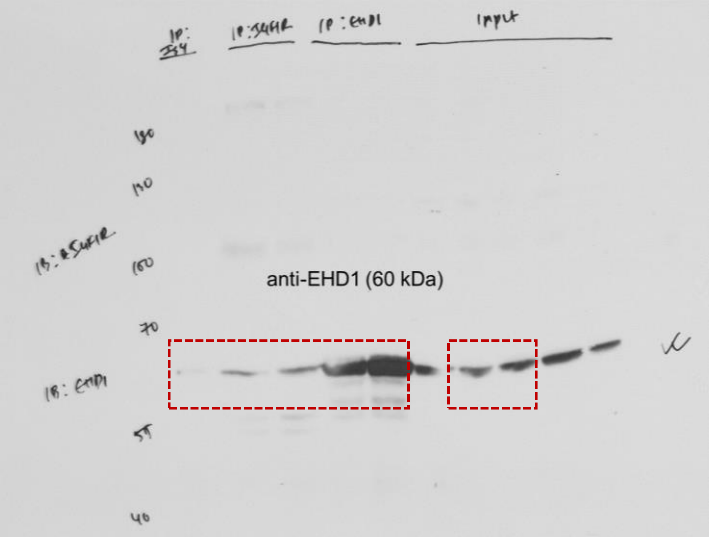

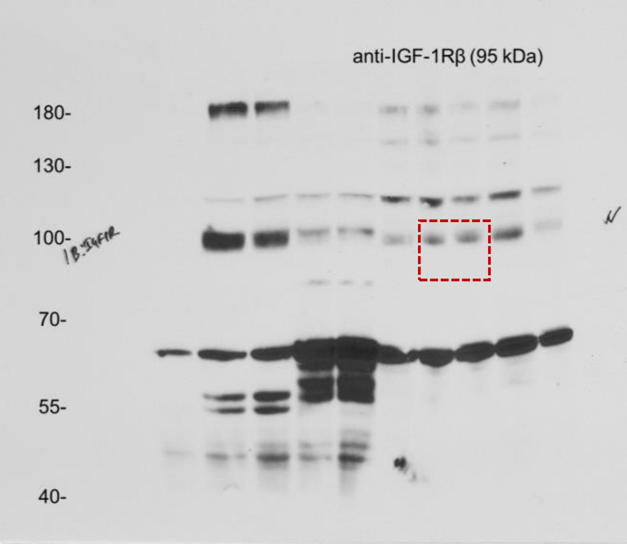

Figure 6

Figure 6a(TC71):

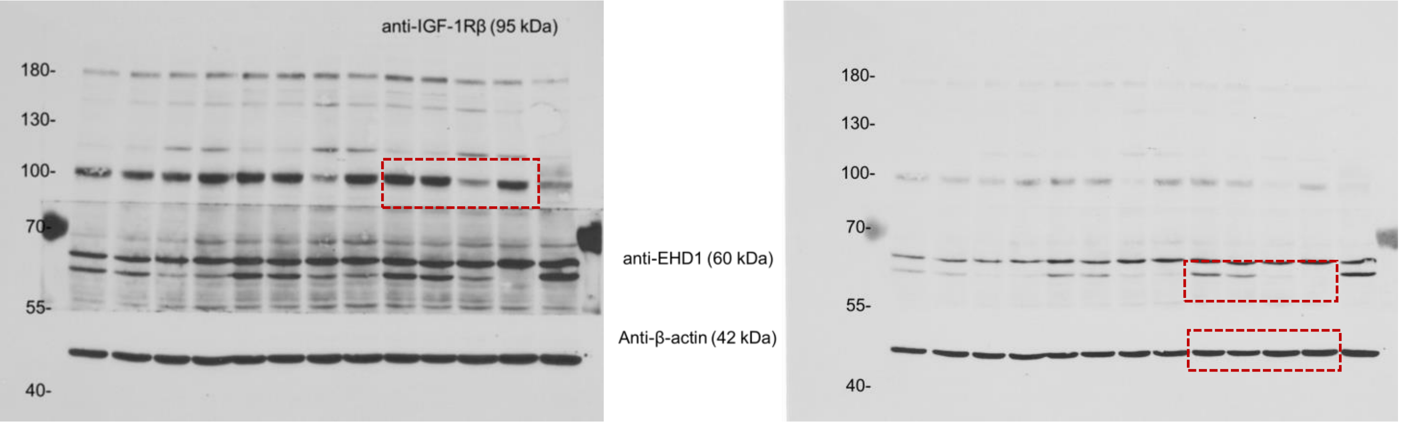

Figure 6a(A673):

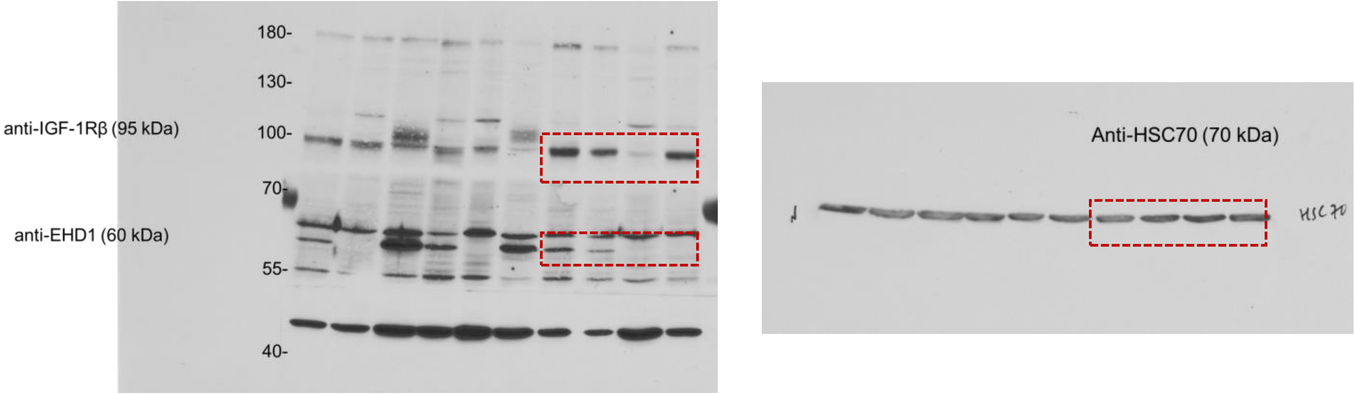

Figure 7

Figure 7a

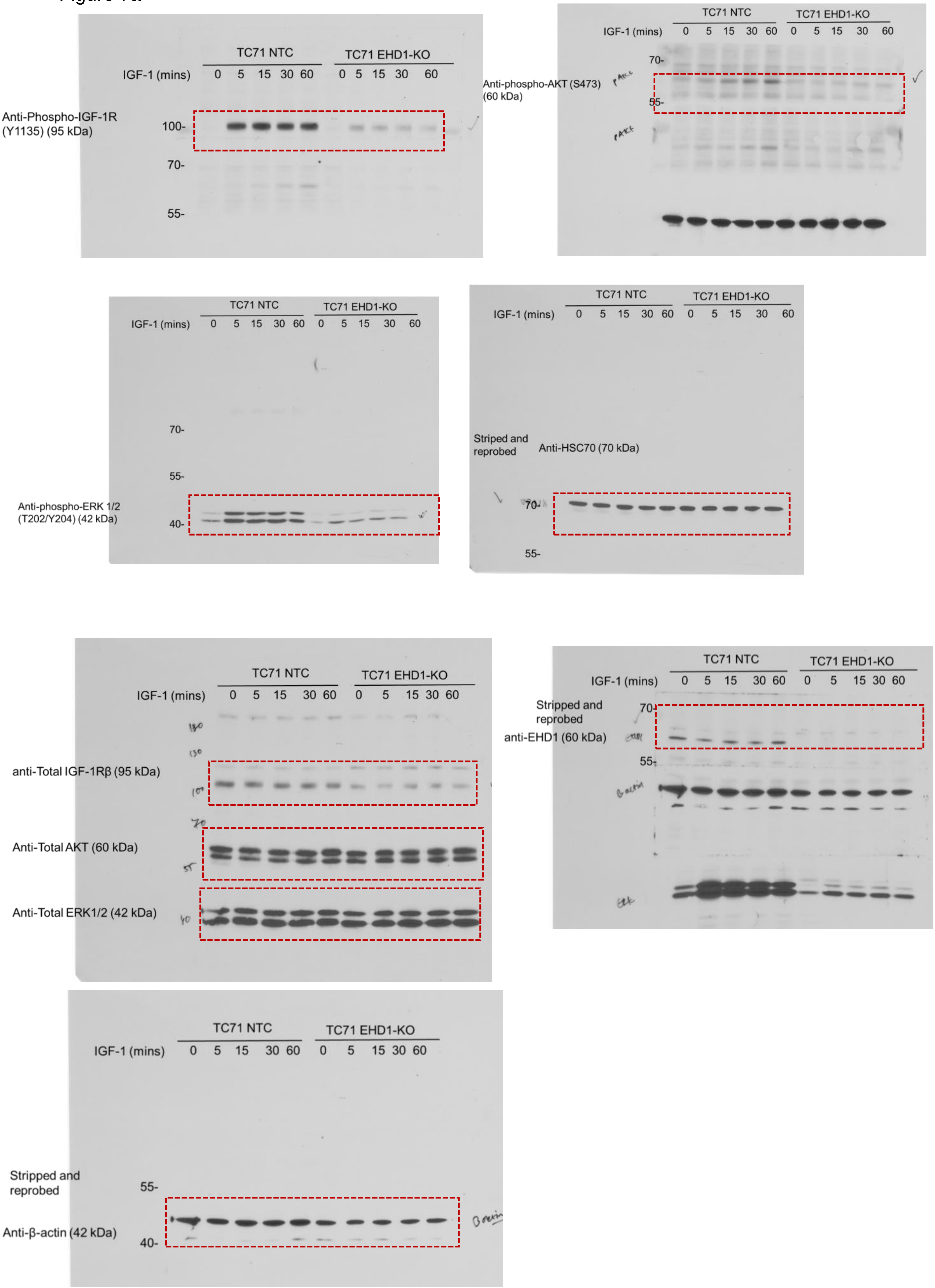

Figure 7

Figure 7b

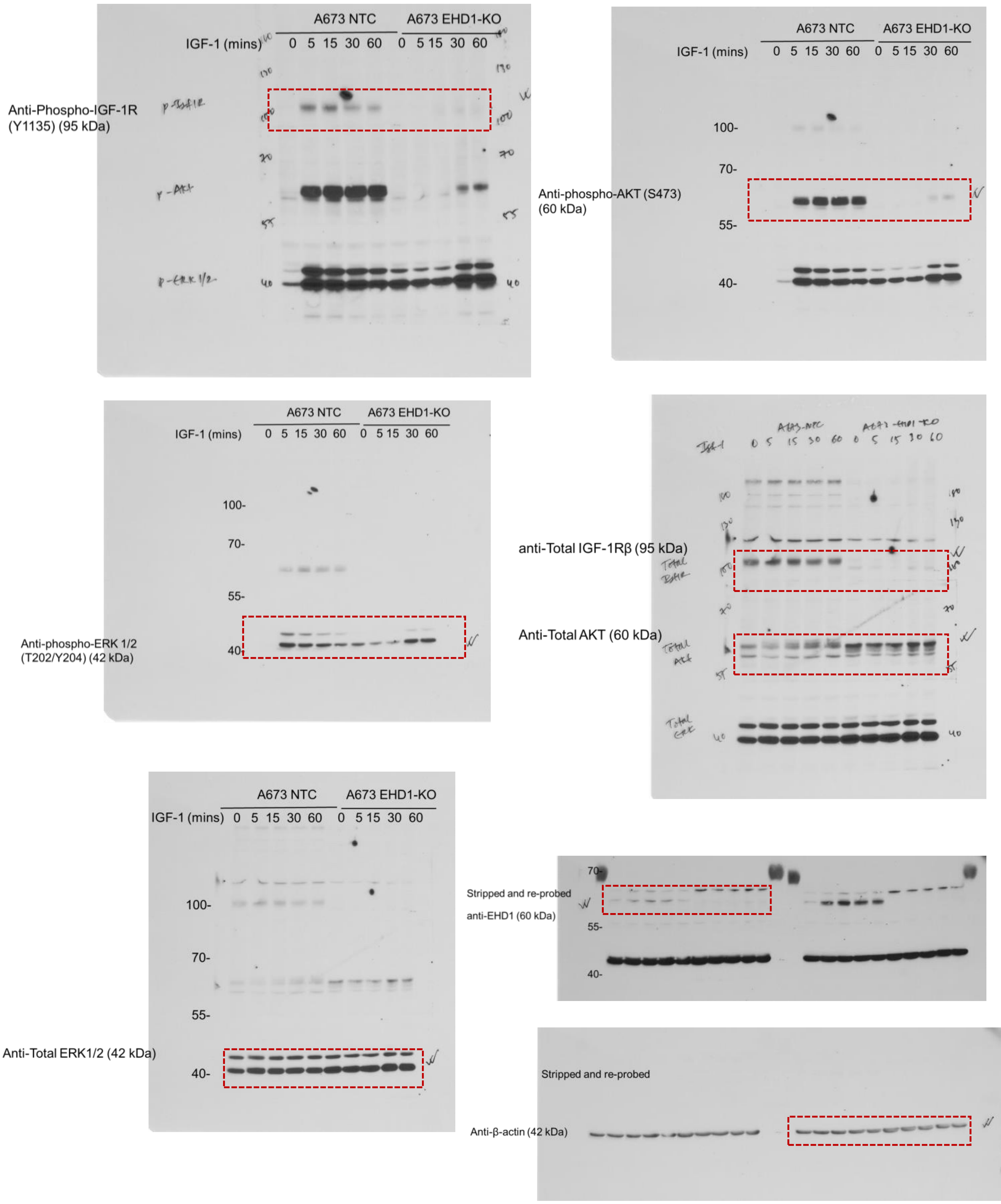

Figure 7

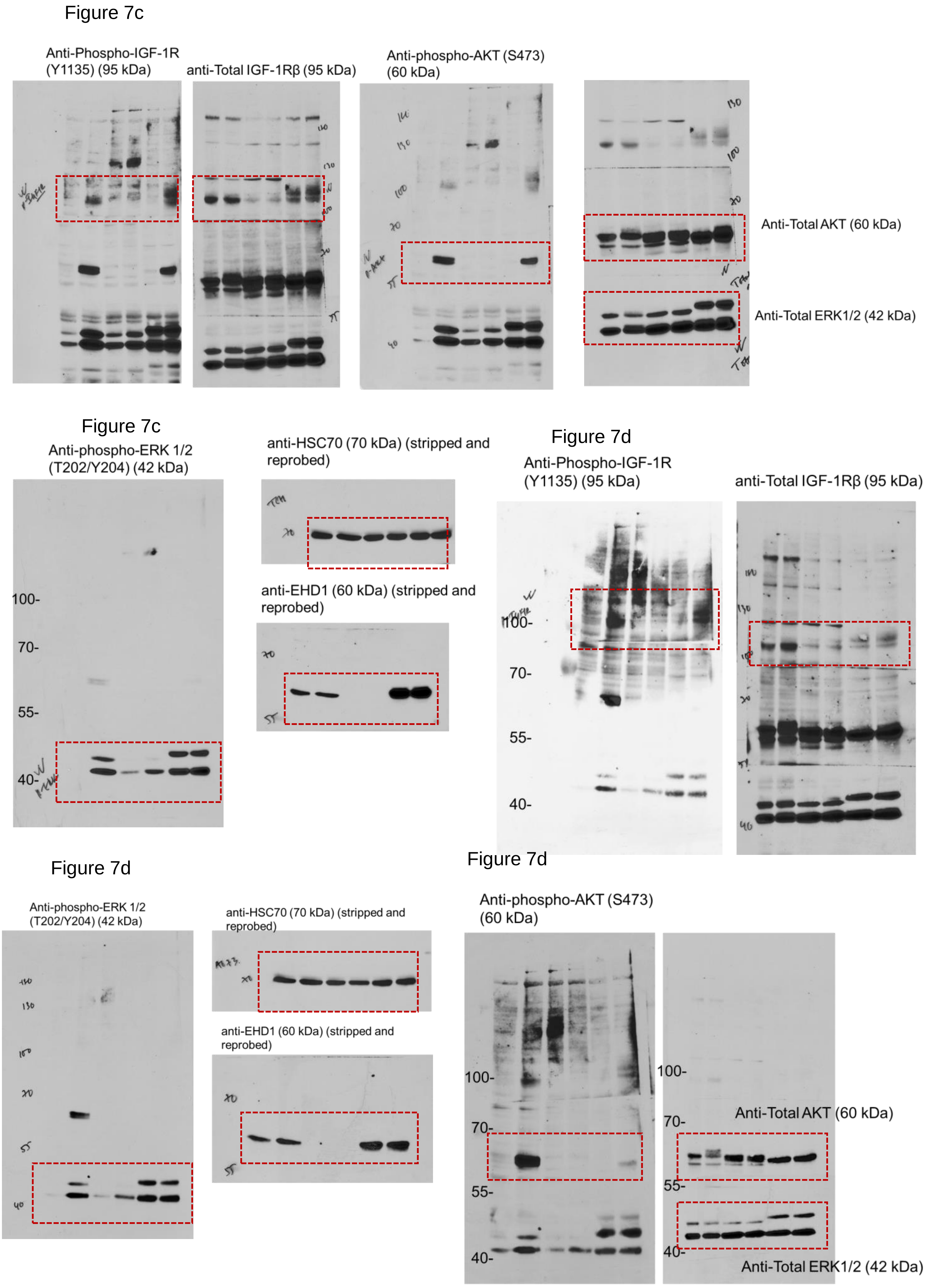
Figure 7c
Anti-Phospho-IGF-1R (Y1135) (95 kDa)
anti-Total IGF-1Rβ (95 kDa)
Anti-phospho-AKT (S473) (60 kDa)
Anti-Total AKT (60 kDa)
Anti-Total ERK1/2 (42 kDa)
Figure 7c
Anti-phospho-ERK 1/2 (T202/Y204) (42 kDa)
100-
70-
55-
40-
anti-HSC70 (70 kDa) (stripped and reprobed)
anti-EHD1 (60 kDa) (stripped and reprobed)
Figure 7d
Anti-Phospho-IGF-1R (Y1135) (95 kDa)
100-
70-
55-
40-
anti-Total IGF-1Rβ (95 kDa)
Figure 7d
Anti-phospho-ERK 1/2 (T202/Y204) (42 kDa)
anti-HSC70 (70 kDa) (stripped and reprobed)
anti-EHD1 (60 kDa) (stripped and reprobed)
Figure 7d
Anti-phospho-AKT (S473) (60 kDa)
100-
70-
55-
40-
100-
70-
55-
40-
Anti-Total AKT (60 kDa)
Anti-Total ERK1/2 (42 kDa)

Figure 7

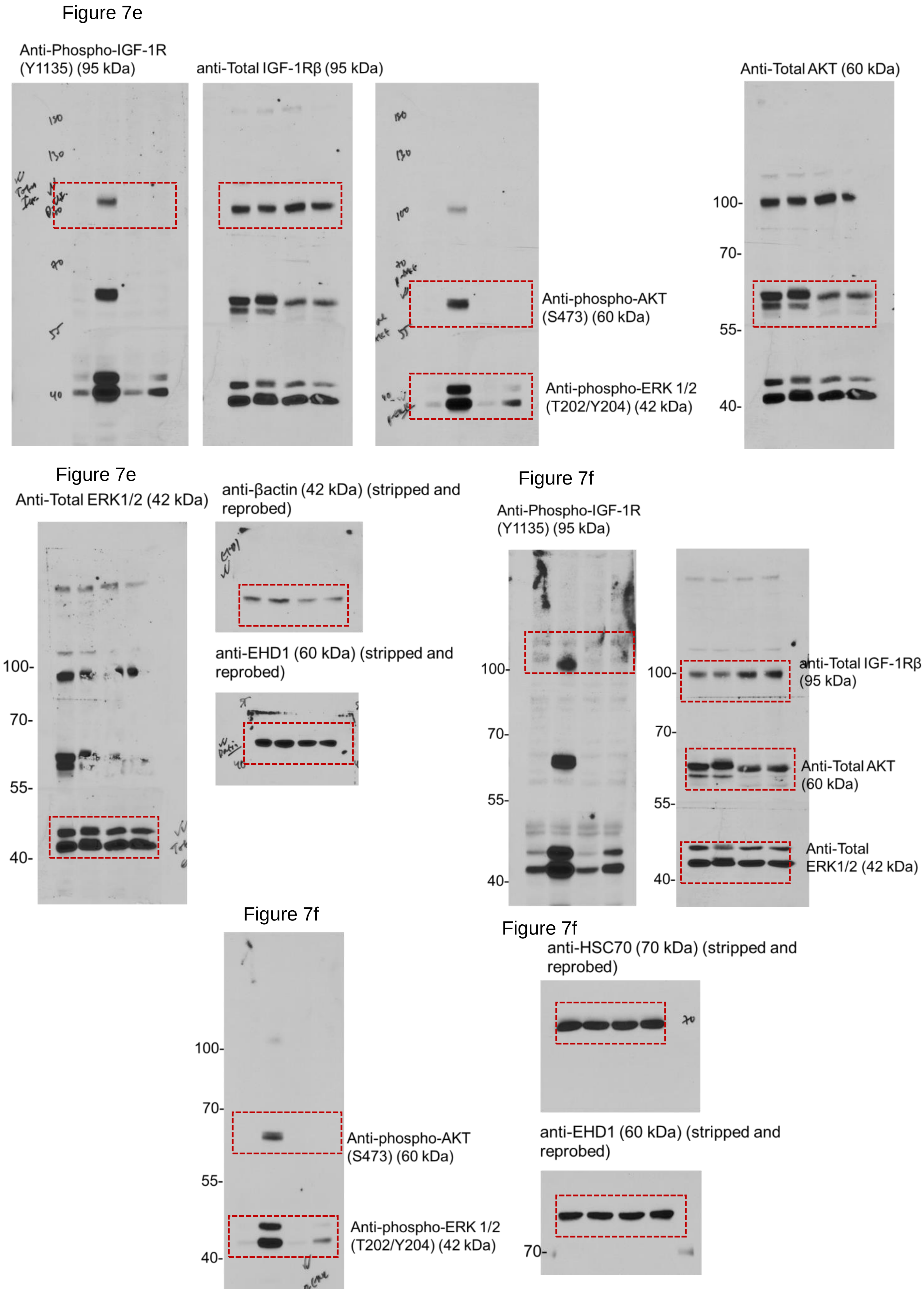
Figure 7e
Anti-Phospho-IGF-1R (Y1135) (95 kDa)
anti-Total IGF-1Rβ (95 kDa)
Anti-phospho-AKT (S473) (60 kDa)
Anti-phospho-ERK 1/2 (T202/Y204) (42 kDa)
Anti-Total AKT (60 kDa)
100-
70-
55-
40-
Figure 7e
Anti-Total ERK1/2 (42 kDa)
100-
70-
55-
40-
anti-βactin (42 kDa) (stripped and reprobed)
anti-EHD1 (60 kDa) (stripped and reprobed)
Figure 7f
Anti-Phospho-IGF-1R (Y1135) (95 kDa)
100-
70-
55-
40-
anti-Total IGF-1Rβ (95 kDa)
Anti-Total AKT (60 kDa)
Anti-Total ERK1/2 (42 kDa)
100-
70-
55-
40-
Figure 7f
100-
70-
55-
40-
Anti-phospho-AKT (S473) (60 kDa)
Anti-phospho-ERK 1/2 (T202/Y204) (42 kDa)
Figure 7f
anti-HSC70 (70 kDa) (stripped and reprobed)
anti-EHD1 (60 kDa) (stripped and reprobed)
70-

Figure 8

Figure 8a

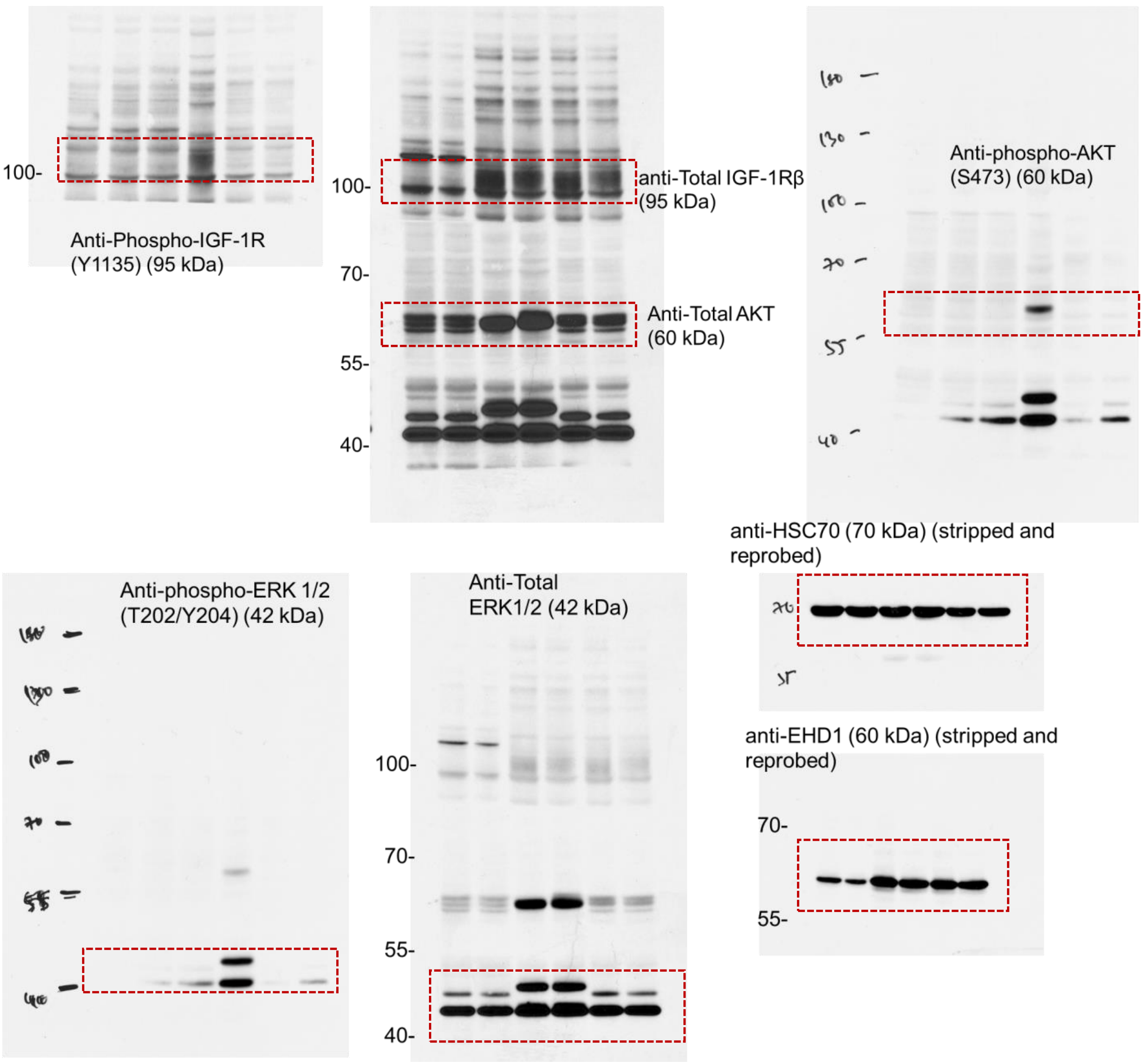

Figure 8b

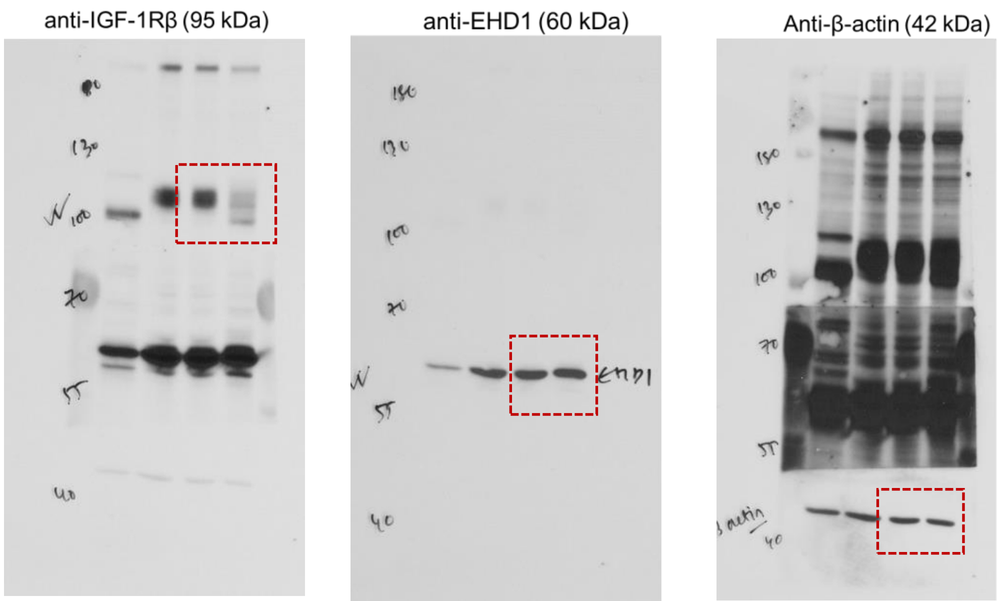

Figure 8

Figure 8j

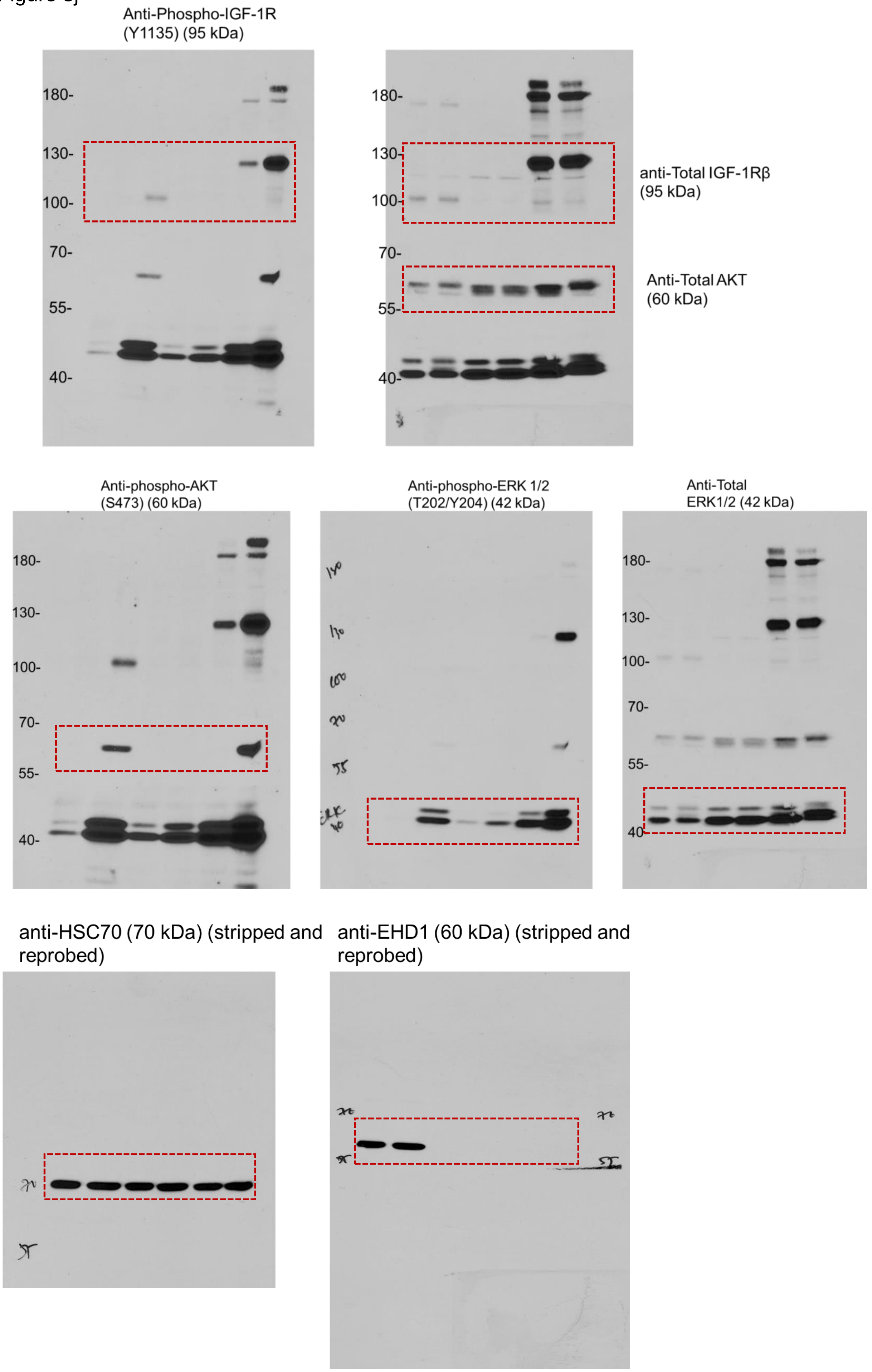

Supplementary Figure S1

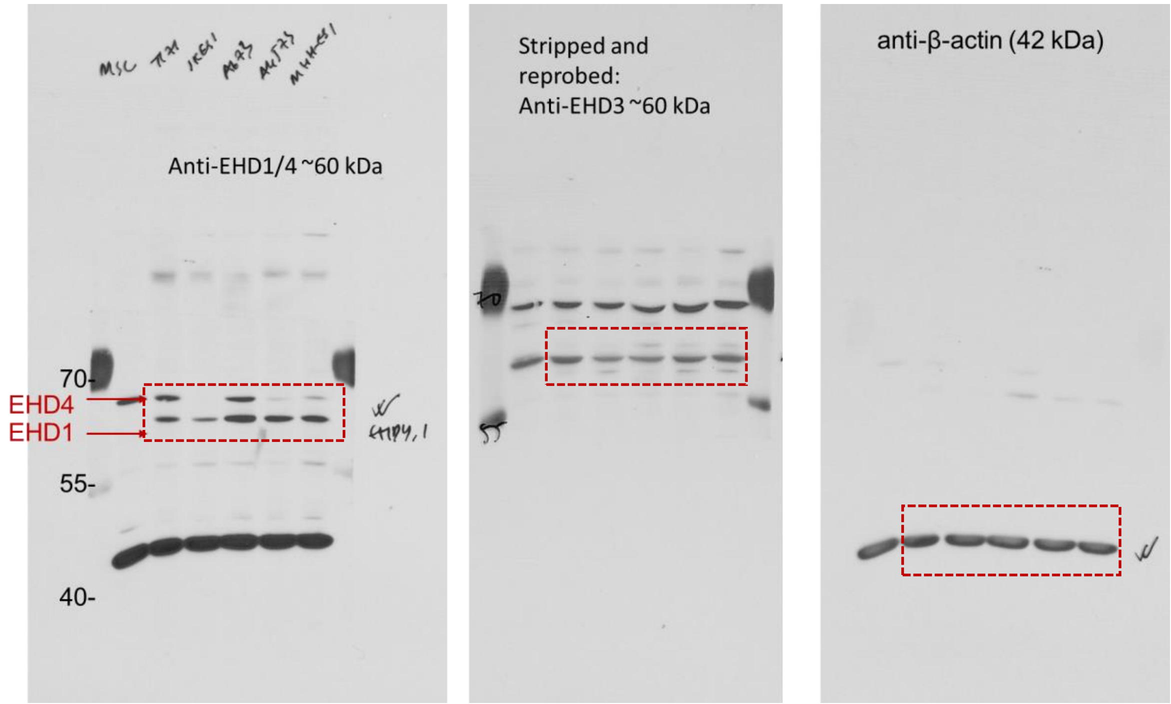

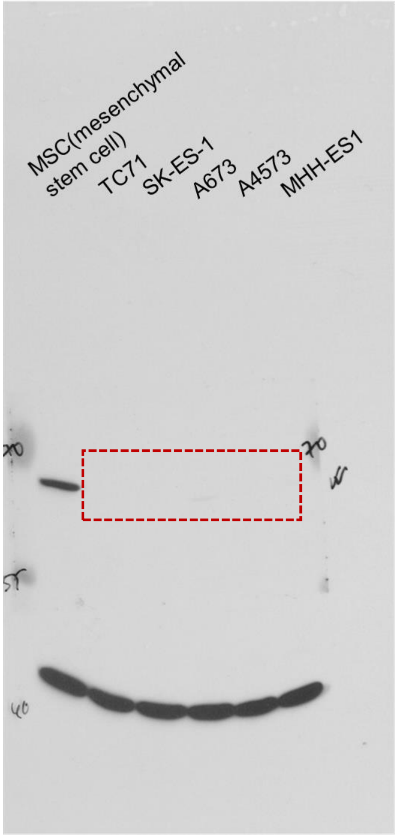

anti-EHD2 (61 kDa)

anti-β-actin (42 kDa)

Supplementary Figure S6

Supplementary Figure S6c

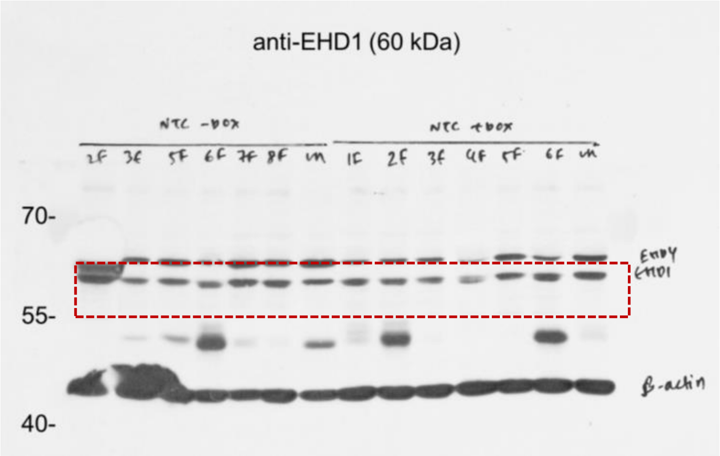

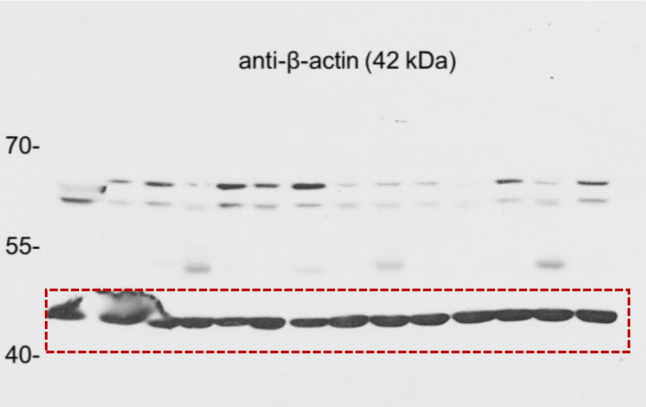

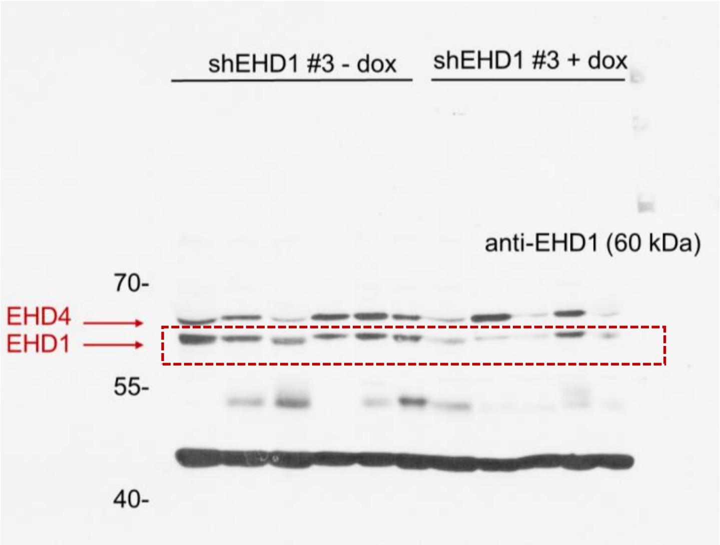

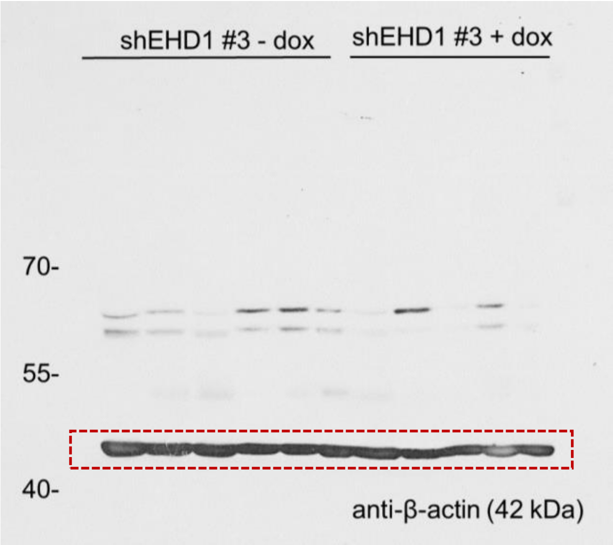

Supplementary Figure S7

Supplementary Figure S7b

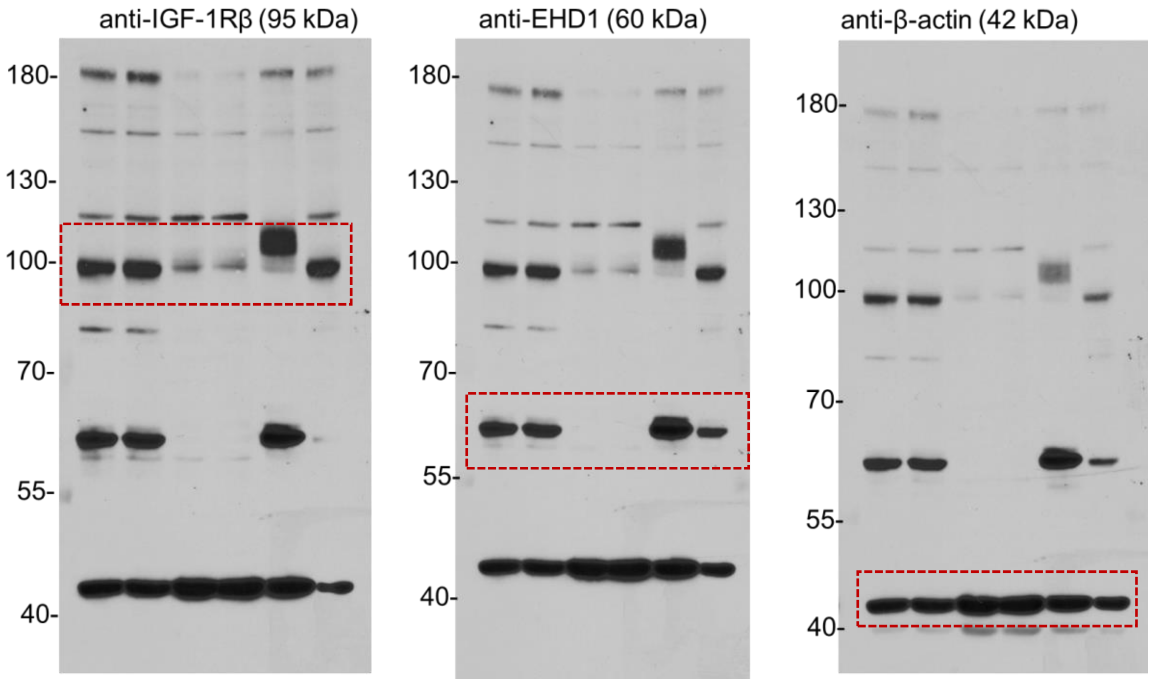

Figure S7c :anti-IGF-1Rβ (95 kDa)

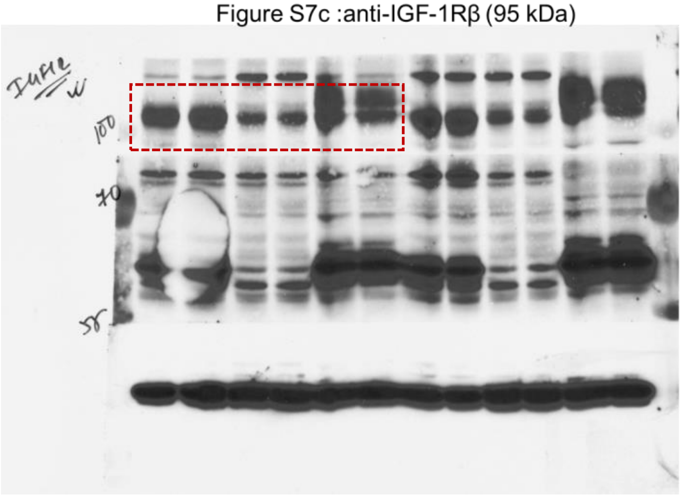

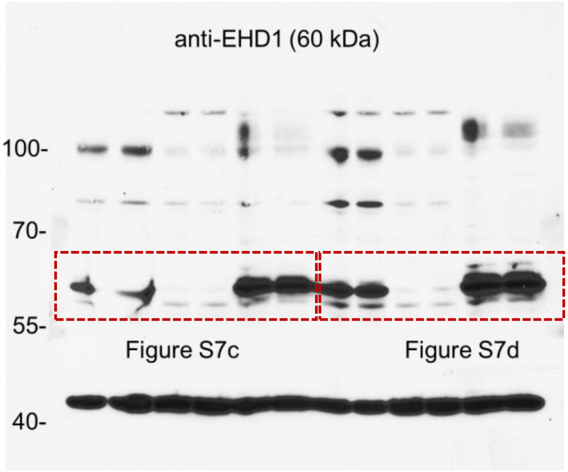

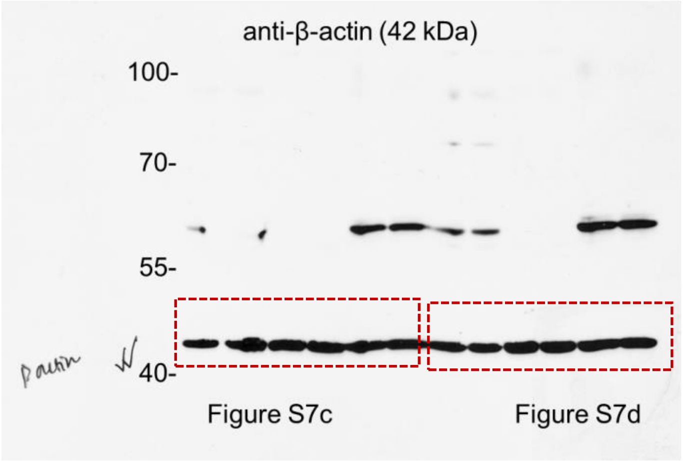

Figure S7d :anti-IGF-1Rβ (95 kDa)

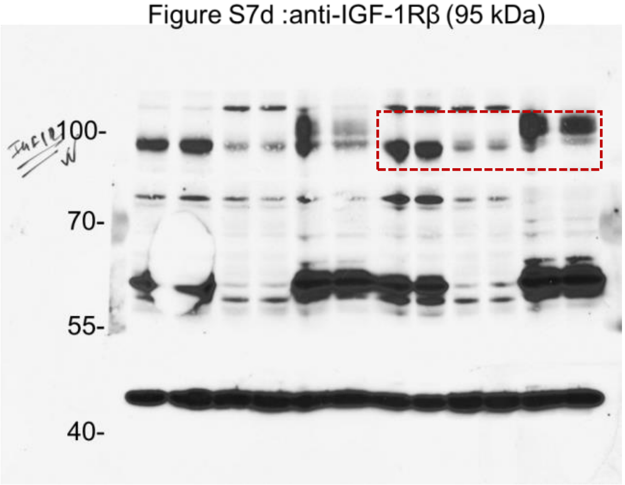

Supplementary Figure S7

Supplementary Figure S7e

anti-IGF-1Rα (130 kDa)

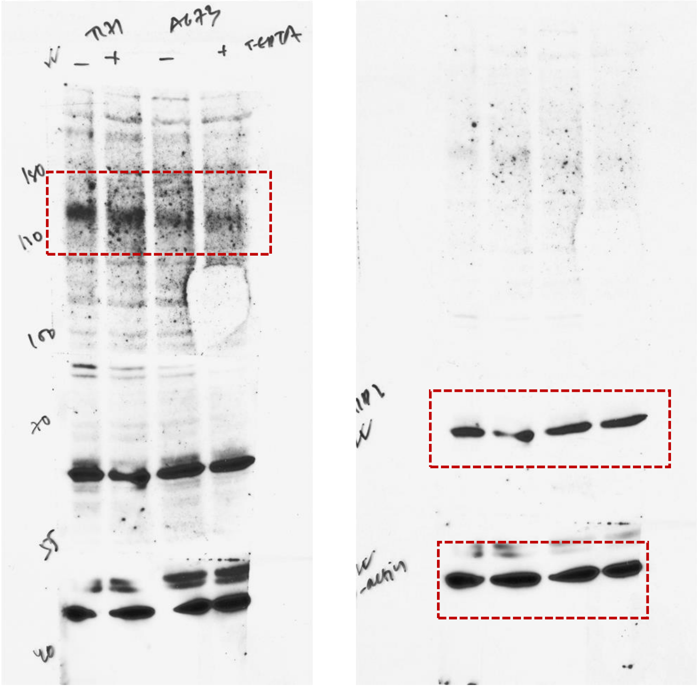

anti-EHD1 (60 kDa)

anti-β-actin (42 kDa)

Supplementary Figure S12

Supplementary Figure S12h

anti-IGF-1Rβ (95 kDa)

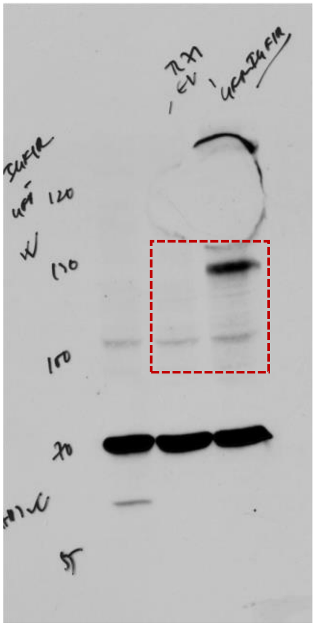

anti-HSC70 (70 kDa)

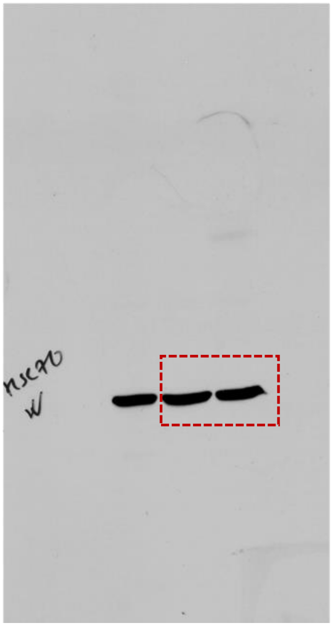

Supplementary Figure S12i

anti-IGF-1Rβ (95 kDa)

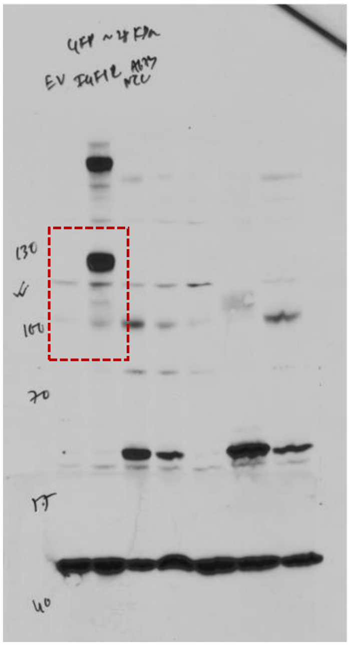

anti-β-actin (42 kDa)

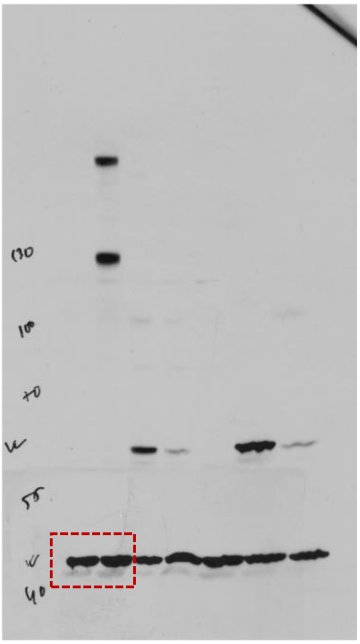

Supplementary Fig. S14

Gating strategy for surface IGF-1R Flow analysis:

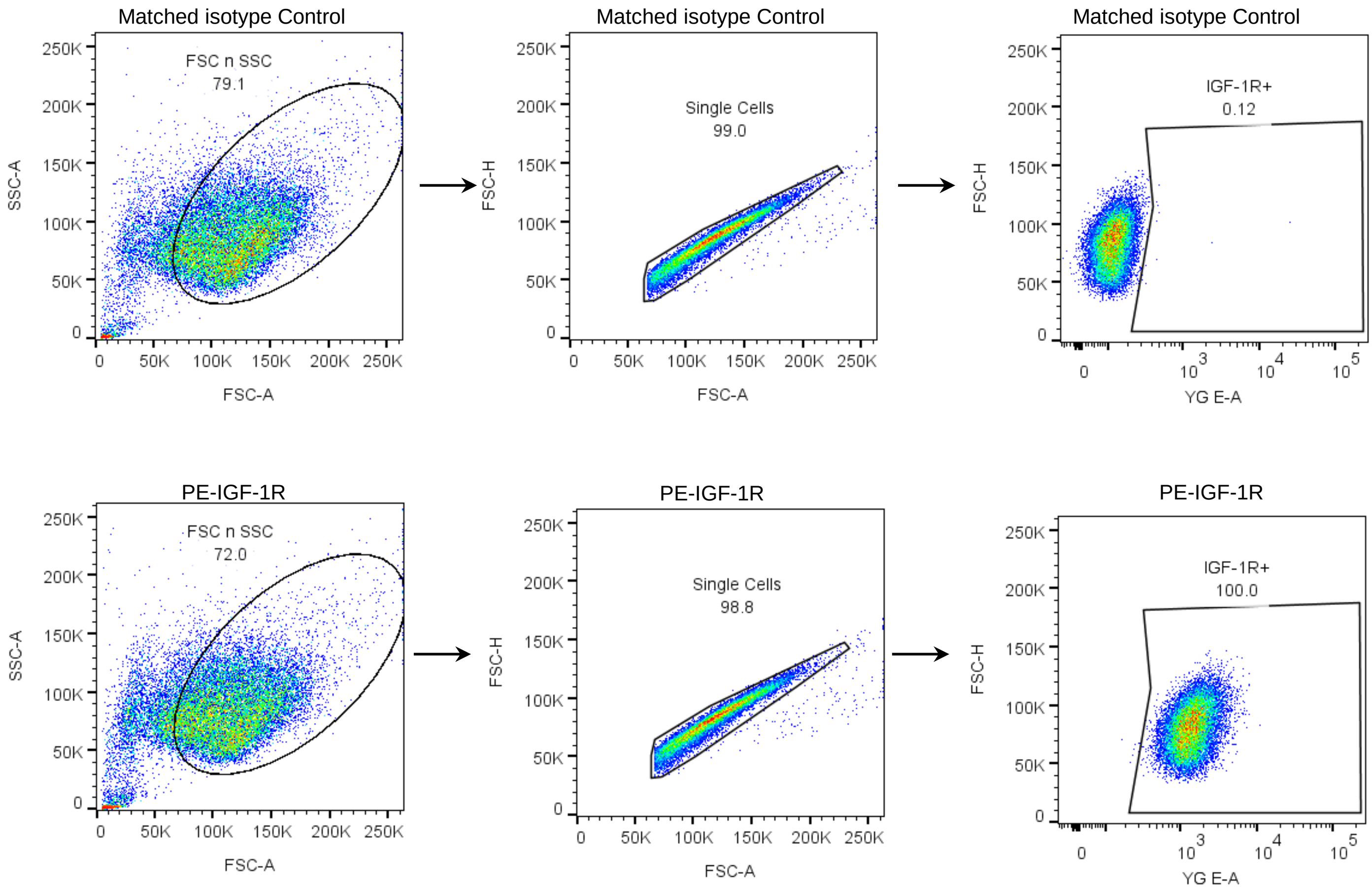

Gating strategy for Annexin-V-PI Flow analysis:

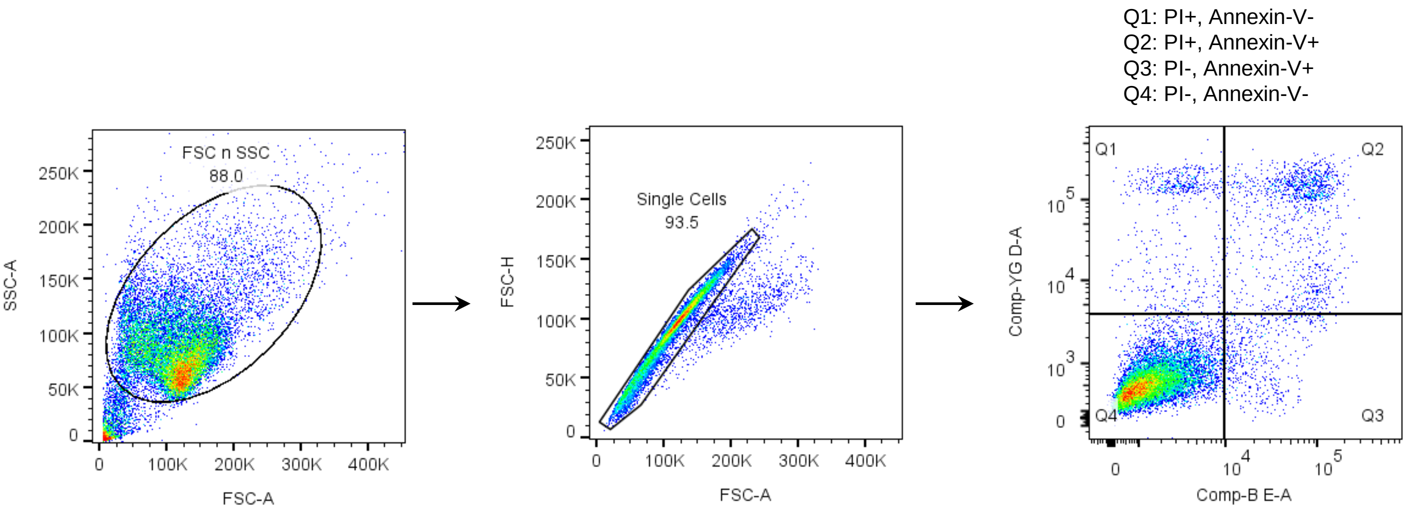

Supplementary Table 1: mRNA expression of EHD1 in Ewing Sarcoma cell lines (CCLE):

RDES	5.098453246
A673	5.095080492
CHLA10	4.716442237
MHHES1	4.697106574
TC71	4.539779192
SKNEP1	4.468583317
EWS502	4.457462965
SKNMC	4.327687364
SKES1	4.213347282
CADOES1	4.183486514
CHLA9	4.04701482
EW8	3.806324057
CHLA32	3.69265037
CBAGPN	3.678071905
CHLA218	3.673556424
CHLA99	3.486714373
TC138	3.416839742
TC205	2.929790998
TC106	2.843983844
Highest of 1,408 cell lines	7.962953828
Lowest of 1,408 cell lines	0.879705766
Median of 1408 cell lines	4.840463234

The CCLE database data are catalogued from RNA-seq files as Log2 TPM (transcripts per million) + 1. Cell lines indicated in blue are used in the current study.

Supplementary Table 2. Higher expression of EHD1 in metastatic lesions:

EHD1	Tissue sample				Total
	Localized	Disseminated	Relapse	Metastasis	
0	33 (12.4%)	0 (0%)	2 (20%)	0 (0%)	35
1	118 (44.2%)	15 (33.3%)	3 (30%)	0 (0%)	126
2	116 (43.4%)	10 (66.7%)	5 (50%)	15 (100%)	146
Total	267	15	10	15	307

$\chi^2 = 22.389$; $p = 0.001$

Spearman's correlation coefficient= 0.211; $p < 0.001$

Supplementary Table 3. Co-expression of IGF-1R-EHD1 - Frequencies considering all tissue types and only primary tumors:

IGF-1R-EHD1		
	All tissues	Primary tumors
Negative	4 (1.9%)	4 (2.2%)
EHD1 or IGF-1R positive	84 (39.1%)	76 (41.5%)
Both positive	127 (59.1%)	103 (56.3%)
Total	183	183

Supplementary Table 4. Correlation between IGF-1R-EHD1 co-expression and tissue types:

IGF-1R_EHD1	Tissue sample				Total
	Localized	Disseminated	Relapse	Metastasis	
Negative	4 (1.9%)	0 (0%)	0 (0%)	0 (0%)	4
EHD1 or IGF-1R+	76 (41.5%)	2 (15.4%)	1 (16.7%)	5 (38.5%)	84
Both positive	103 (56.3%)	11 (84.6%)	5 (83.3%)	8 (61.5%)	127
Total	183	13	6	13	215

Supplementary Table 5. Association between IGF-1R-EHD1 co-expression and Overall survival (OS):

Parameters	n	Events	%OS	p-Univariate
IGF-1R_EHD1				
Negative	4	1	75%	0.600
EHD1 or IGF-1R+	66	35	42%	
Both positive	91	44	41%	

Supplementary Table 6. Association between IGF-1R-EHD1 co-expression and Progression free survival (PFS):

Parameters	n	Events	%PFS	p-Univariate
IGF-1R_EHD1				
Negative	4	1	75%	0.334
EHD1 or IGF-1R +	66	38	34.4%	
Both positive	92	43	47%	

Supplementary Table 7. Correlation between IGF-1R and EHD1 IHC expression (4 categories):

	EHD1				Total
IGF-1R	0	1	2	3	
0	0 (0%)	2 (28.6%)	21 (22.6%)	9 (8%)	32
1	0 (0%)	2 (28.6%)	22 (23.7%)	27 (23.9%)	51
2	1 (50%)	3 (42.9%)	42 (45.2%)	60 (53.1%)	106
3	1 (50%)	0 (0%)	8 (8.6%)	17 (15%)	26
Total	2	7	93	113	215

χ^2=14.747; p=0.098

Spearman's Correlation coefficient= 0.179; p=0.009
