# Supplementary material for: EHD1-dependent traffic of IGF-1 receptor to the cell surface is essential for Ewing sarcoma tumorigenesis and metastasis
Source: Commun Biol. 2023 Jul 20;6:758. doi: 10.1038/s42003-023-05125-1 (PMC10359273; doi:10.1038/s42003-023-05125-1)
Supplement: Supplementary file 3 — Description of Additional Supplementary Files [file 42003_2023_5125_MOESM3_ESM.pdf]

Description of Additional Supplementary Files

File name: Supplementary Data
Description: Numerical source data for graphs and charts.
