# Supplementary material for: EHD1-dependent traffic of IGF-1 receptor to the cell surface is essential for Ewing sarcoma tumorigenesis and metastasis
Source: Commun Biol. 2023 Jul 20;6:758. doi: 10.1038/s42003-023-05125-1 (PMC10359273; doi:10.1038/s42003-023-05125-1)
Supplement: Supplementary file 5 — Reporting Summary [file 42003_2023_5125_MOESM5_ESM.pdf]

nature portfolio

Corresponding author(s): Bhopal C. Mohapatra, Hamid Band

Last updated by author(s): Jul 5, 2023

Reporting Summary

Nature Portfolio wishes to improve the reproducibility of the work that we publish. This form provides structure for consistency and transparency in reporting. For further information on Nature Portfolio policies, see our Editorial Policies and the Editorial Policy Checklist.

Statistics

For all statistical analyses, confirm that the following items are present in the figure legend, table legend, main text, or Methods section.

n/a	Confirmed	
☐	☒	The exact sample size (*n*) for each experimental group/condition, given as a discrete number and unit of measurement
☐	☒	A statement on whether measurements were taken from distinct samples or whether the same sample was measured repeatedly
☐	☒	The statistical test(s) used AND whether they are one- or two-sided<br>*Only common tests should be described solely by name; describe more complex techniques in the Methods section.*
☐	☒	A description of all covariates tested
☐	☒	A description of any assumptions or corrections, such as tests of normality and adjustment for multiple comparisons
☐	☒	A full description of the statistical parameters including central tendency (e.g. means) or other basic estimates (e.g. regression coefficient) AND variation (e.g. standard deviation) or associated estimates of uncertainty (e.g. confidence intervals)
☒	☐	For null hypothesis testing, the test statistic (e.g. *F*, *t*, *r*) with confidence intervals, effect sizes, degrees of freedom and *P* value noted<br>*Give P values as exact values whenever suitable.*
☒	☐	For Bayesian analysis, information on the choice of priors and Markov chain Monte Carlo settings
☒	☐	For hierarchical and complex designs, identification of the appropriate level for tests and full reporting of outcomes
☒	☐	Estimates of effect sizes (e.g. Cohen's *d*, Pearson's *r*), indicating how they were calculated

Our web collection on statistics for biologists contains articles on many of the points above.

Software and code

Policy information about availability of computer code

Data collection	not relevant to our study
Data analysis	not relevant to our study

For manuscripts utilizing custom algorithms or software that are central to the research but not yet described in published literature, software must be made available to editors and reviewers. We strongly encourage code deposition in a community repository (e.g. GitHub). See the Nature Portfolio guidelines for submitting code & software for further information.

Data

Policy information about availability of data

All manuscripts must include a data availability statement. This statement should provide the following information, where applicable:

- Accession codes, unique identifiers, or web links for publicly available datasets
- A description of any restrictions on data availability
- For clinical datasets or third party data, please ensure that the statement adheres to our policy

RNA-seq data have been deposited at the Gene Expression Omnibus (GEO; GSE225214) with a link for reviewers to access at: https://www.ncbi.nlm.nih.gov/geo/query/acc.cgi?acc=GSE225214 the secure token "kloxomyqrxapvsn"

Human research participants

Policy information about studies involving human research participants and Sex and Gender in Research.

Reporting on sex and gender	No direct involvement with human research participants. A total of 324 paraffin-embedded TMA samples from ESFT patients from the period comprised between April 1971 and May 2007 treated at Instituto Ortopedici Rizzoli (IOR), Bologna, Italy, and at the Department of Pathology of the University of Valencia Estudi General (UVEG), Spain were analyzed within the context of two European Translational Research projects [PROTHETS (http://www.prothets.org) and EuroBo-Net (http://www.eurobonet.eu)]. Characteristics of the cohort and relevant clinical information have been previously reported (cited in manuscript).
Population characteristics	Characteristics of the cohort and relevant clinical information have been previously reported (cited in manuscript).
Recruitment	Characteristics of the cohort and relevant clinical information have been previously reported (cited in manuscript).
Ethics oversight	Approval for data acquisition and analysis was obtained from the Ethics Committee of the institutions involved in the study.

Note that full information on the approval of the study protocol must also be provided in the manuscript.

Field-specific reporting

Please select the one below that is the best fit for your research. If you are not sure, read the appropriate sections before making your selection.

☒ Life sciences ☐ Behavioural & social sciences ☐ Ecological, evolutionary & environmental sciences

For a reference copy of the document with all sections, see nature.com/documents/nr-reporting-summary-flat.pdf

Life sciences study design

All studies must disclose on these points even when the disclosure is negative.

Sample size	Sample sizes were determined by power analysis. For mouse xenografts: Using a t-test, a sample size of n=6 per group provided 80% power with a two-sided alpha=0.05 to detect an effect size d=1.78 standard deviations (assuming a common group standard deviation). For cell line studies, a sample size of n=3 provided 80% power at the 0.05 level of significance (two-sided) to detect a standardized effect size of 3.26.
Data exclusions	No data were excluded from analysis.
Replication	All attempts in replication were successful.
Randomization	Not relevant to our study.
Blinding	Not relevant to our study.

Reporting for specific materials, systems and methods

We require information from authors about some types of materials, experimental systems and methods used in many studies. Here, indicate whether each material, system or method listed is relevant to your study. If you are not sure if a list item applies to your research, read the appropriate section before selecting a response.

Materials & experimental systems

n/a	Involved in the study	
☐	☒	Antibodies
☐	☒	Eukaryotic cell lines
☒	☐	Palaeontology and archaeology
☐	☒	Animals and other organisms
☒	☐	Clinical data
☒	☐	Dual use research of concern

Methods

n/a	Involved in the study	
☒	☐	ChIP-seq
☐	☒	Flow cytometry
☒	☐	MRI-based neuroimaging

Antibodies

Antibodies used	Primary antibodies used for immunoblotting were as follows: anti-HSC70 (#sc-7298) from Santa Cruz Biotechnology; anti-IGF-1Rβ

Antibodies used	(#3018), anti-phospho-IGF-1R-Y1135 (#3918), anti-phospho-AKT-S473 (#4060), anti-AKT (#4685), anti-ERK1/2 (#4695), anti-phospho-ERK1/2- Thr202/Tyr204 (#9101) from Cell Signaling Technology; and anti-beta-actin (#A5441) from Sigma. In-house generated Protein G-purified rabbit polyclonal anti-EHD1, EHD2, EHD3 and EHD4 antibodies have been described previously7. The horseradish peroxidase (HRP)-conjugated Protein A (#101023) and HRP-conjugated rabbit anti-mouse secondary antibody (#31430) for immunoblotting were from Thermo Fisher. Antibodies used for immunofluorescence studies were as follows: anti-EHD1 (#ab109311) from Abcam; Alexa-555-conjugated anti-GM130 (#48641), anti-LAMP1 (#9091) and anti-RAB11 (#5589) from Cell Signaling Technology; and anti-IGF-1Rβ (#MA5-13802) from Invitrogen. Secondary antibodies used for immunofluorescence studies were Alexa Fluor 594-conjugated goat anti-rabbit IgG (H + L) (#A11012) or Alexa Fluor 488-conjugated goat anti-mouse IgG (H + L) (#A11001) from Life Technologies Corporation. The Annexin-V-PI flow cytometric analysis was done using a kit (#V13241) from Invitrogen. Primary antibodies used for immunohistochemical studies included: anti-IGF-1R (#14534) and anti-cleaved-caspase 3 (#9661) from Cell Signaling Technology; and anti-CD99 (#ab-227738) and anti-Ki67 (#ab92353) from Abcam. For immunoprecipitation studies, primary antibodies included: anti-IGF-1Rβ (Cell Signaling technology; #9750), anti-EHD1 (Abcam; #ab109311) and anti-Rabbit-IgG (Invitrogen; #02-6102).
Validation	Antibodies were validated in-house in multiple research applications by using control cell lines, siRNAs and used according to manufacturer's instructions.

Eukaryotic cell lines

Policy information about cell lines and Sex and Gender in Research

Cell line source(s)	Human Ewing Sarcoma cell lines TC-71, MHH-ES-1 and A4573 were obtained from Dr. Jason Yustein laboratory at Baylor college of medicine(TC-71, MHH-ES-1:DSMZ-German collection, A4573: Cellonco). A673 and SK-ES-1 cells were obtained from ATCC. HEK-293T cells were from ATCC (CRL-3216).
Authentication	Cell lines from ATCC were authenticated by their cell line authentication service. Cell lines not obtained from the American Type Culture Collection were verified by genetic analysis performed by the UNMC Molecular Diagnostics Laboratory.
Mycoplasma contamination	Cell lines were maintained for less than 30 days in continuous culture and were regularly tested negative for mycoplasma.
Commonly misidentified lines (See ICLAC register)	n/a

Animals and other research organisms

Policy information about studies involving animals; ARRIVE guidelines recommended for reporting animal research, and Sex and Gender in Research

Laboratory animals	6-week-old female and male athymic nude mice
Wild animals	n/a
Reporting on sex	Both female and male mice were used.
Field-collected samples	n/a
Ethics oversight	All animal experiments were performed with the approval of the UNMC Institutional Animal Care and Use Committee (IACUC Protocol 19-017-04-FC).

Note that full information on the approval of the study protocol must also be provided in the manuscript.

Flow Cytometry

Plots

Confirm that:

☒ The axis labels state the marker and fluorochrome used (e.g. CD4-FITC).

☒ The axis scales are clearly visible. Include numbers along axes only for bottom left plot of group (a 'group' is an analysis of identical markers).

☒ All plots are contour plots with outliers or pseudocolor plots.

☒ A numerical value for number of cells or percentage (with statistics) is provided.

Methodology

Sample preparation	2x105 cells were seeded per well of six-well plates and grown in regular medium with 10% FBS for 48h. Cells were further treated as indicated in figure legends, rinsed with ice-cold PBS, released from dishes with trypsin-EDTA (#15400054; LifeTech (ThermoFisher)) and the trypsinization stopped by adding equal volume of soybean trypsin inhibitor (#17075029; LifeTech (ThermoFisher) Cells were washed thrice in ice-cold FACS buffer (1% BSA in PBS), and live cells stained with PE-anti-human-IGF-1R (#351806; Biolegend) or PE-Mouse-IgG isotype control (#400112; Biolegend).

Instrument	FACS analyses were performed on a LSRFortessa X50 instrument
Software	Data were analyzed using the FlowJo software.
Cell population abundance	n/a
Gating strategy	Preliminary gates: FSC n SSC, single cell population, then positive/negative cell populations were defined by PE-Mouse-IgG isotype control used for each cell type and replicate separately stained with PE-anti-human-IGF-1R.

☒ Tick this box to confirm that a figure exemplifying the gating strategy is provided in the Supplementary Information.
